# Characterization of a Bacterial Kinase That Phosphorylates Dihydrosphingosine to Form dhS1P

**DOI:** 10.1128/spectrum.00002-22

**Published:** 2022-03-14

**Authors:** Dev K. Ranjit, Zachary D. Moye, Fernanda G. Rocha, Gregory Ottenberg, Frank C. Nichols, Hey-Min Kim, Alejandro R. Walker, Frank C. Gibson, Mary E. Davey

**Affiliations:** a Department of Oral Biology, College of Dentistry, University of Florida, Gainesville, Florida, USA; b Division of Periodontology, University of Connecticut School of Dental Medicine, Farmington, Connecticut, USA; Ohio State University

**Keywords:** sphingolipids, dihydrosphingosine, sphinganine, sphingosine kinase, dihydroceramides, *Porphyromonas gingivalis*, *Bacteroidetes*, inflammation, macrophages

## Abstract

Like other members of the phylum *Bacteroidetes*, the oral anaerobe Porphyromonas gingivalis synthesizes a variety of sphingolipids, similar to its human host. Studies have shown that synthesis of these lipids (dihydroceramides [DHCs]) is involved in oxidative stress resistance, the survival of P. gingivalis during stationary phase, and immune modulation. Here, we constructed a deletion mutant of P. gingivalis strain W83 with a deletion of the gene encoding DhSphK1, a protein that shows high similarity to a eukaryotic sphingosine kinase, an enzyme that phosphorylates sphingosine to form sphingosine-1-phosphate. Our data show that deletion of the dhSphK1 gene results in a shift in the sphingolipid composition of P. gingivalis cells; specifically, the mutant synthesizes higher levels of phosphoglycerol DHCs (PG-DHCs) than the parent strain W83. Although PG1348 shows high similarity to the eukaryotic sphingosine kinase, we discovered that the PG1348 enzyme is unique, since it preferentially phosphorylates dihydrosphingosine, not sphingosine. Besides changes in lipid composition, the W83 ΔPG1348 mutant displayed a defect in cell division, the biogenesis of outer membrane vesicles (OMVs), and the amount of K antigen capsule. Taken together, we have identified the first bacterial dihydrosphingosine kinase whose activity regulates the lipid profile of P. gingivalis and underlies a regulatory mechanism of immune modulation.

**IMPORTANCE** Sphingoid base phosphates, such as sphingosine-1-phosphate (S1P) and dihydrosphingosine-1-phosphate (dhS1P), act as ligands for S1P receptors, and this interaction is known to play a central role in mediating angiogenesis, vascular stability and permeability, and immune cell migration to sites of inflammation. Studies suggest that a shift in ratio to higher levels of dhS1P in relation to S1P alters downstream signaling cascades due to differential binding and activation of the various S1P receptor isoforms. Specifically, higher levels of dhS1P are thought to be anti-inflammatory. Here, we report on the characterization of a novel kinase in Porphyromonas gingivalis that phosphorylates dihydrosphingosine to form dhS1P.

## INTRODUCTION

The human oral cavity is inhabited by an array of microbiota, including several species known to be proficient in sphingolipid biosynthesis, such as Porphyromonas gingivalis, Tannerella forsythia, and Prevotella intermedia (1–3). P. gingivalis is strongly implicated in the progression of periodontal disease (4), a common biofilm-based chronic inflammatory disease of humans (5). The ability of P. gingivalis to persist within the oral cavity and contribute to chronic infection is attributed to a unique repertoire of virulence determinants, including a capsule (6), atypical lipopolysaccharides (LPS) (7), and a host of proteases and protein-modifying enzymes (reviewed in references 8 and 9). And yet, precisely how P. gingivalis may influence and contribute to the changing dynamics of the inflammatory pathways as certain *in vivo* sites progress from gingivitis to periodontitis is not clear. For this reason, a key area of focus has been to determine how endogenous pathogens like P. gingivalis can promote a lesion that does not heal.

Encapsulation is a well-known mechanism that protects bacteria from clearance by host immune defenses (10). This reduction in clearance can lead to persistent survival and, thereby, long-term interplay between the bacterium and host. Not only do capsules reduce the ability of the host effectors to gain access to the bacterial cell, but they can also mask the cell surface and play a role in immune evasion (11). Strains of P. gingivalis that produce a capsule are more resistant to phagocytosis (12), resulting in dissemination and systemic infections in a murine lesion model (13). Interestingly, studies have shown that purified K-1 and K-2 capsule elicit chemokine production from phagocytic cells (14), suggesting that the host response to this antigen may contribute to the formation of a chronic inflammatory lesion. Although it is becoming evident that the synthesis of K antigen capsule is an important virulence determinant (15), its involvement and the role of other surface glycans (A-LPS and O-LPS) in the overall deregulation of host responses is not fully understood.

In eukaryotes, sphingolipids not only serve as structural components of membranes, they also form lipid rafts, providing energetically favorable microdomains capable of stabilizing a variety of membrane proteins (16). In addition, sphingolipid metabolites, such as ceramide, sphingosine-1-phosphate (S1P), and dihydrosphingosine-1-phosphate (dhS1P), are pivotal regulators of numerous signaling pathways (17). Importantly, sphingolipids are only synthesized by a select few genera of prokaryotes and most of these bacteria inhabit a mammalian host (18–24). Like the sphingolipids of other members of the *Bacteroidetes*, the sphingolipids synthesized by P. gingivalis are similar to those produced by eukaryotes, with notable differences. While the core of the sphingoid base in eukaryotes is sphingosine containing a double bond between C-4 and C-5, the sphingoid base of the bacterial sphingolipids is fully saturated and therefore referred to as dihydrosphingosine. In addition, the terminal chain bears an *iso* branch (2). Although studies to determine their function are limited, evidence is accumulating that bacterial sphingolipids serve an important role in protecting sphingolipid-producing bacteria from oxidative stress and survival during stationary phase, and these lipids also appear to form membrane microdomains, similarly to their eukaryotic counterparts (18, 22, 25). In regard to P. gingivalis, we have shown that sphingolipid synthesis correlates with the presentation of K antigen capsule and A-LPS (22) and with the organism’s ability to minimize an inflammatory response (26). Overall, studies are showing that the synthesis of these host-like lipids influences the basic physiology and the pathogenic potential of P. gingivalis. Analysis of P. gingivalis lipid extracts revealed that P. gingivalis cells contain both nonphosphorylated “free” dihydroceramides (DHCs) and phosphorylated dihydroceramides. The major phosphorylated dihydroceramides are identified as having substitutions of phosphoglycerol to form phosphoglycerol dihydroceramide (PG-DHC) or of phosphoethanolamine to form phosphoethanolamine dihydroceramide (PE-DHC). Among the pool of mature sphingolipids synthesized by P. gingivalis, each individual component has been shown to stimulate distinct biological activities in different cell types (2). Importantly, the biochemical pathway for synthesis of the sphingolipids has not yet been elucidated.

**FIG 1:**
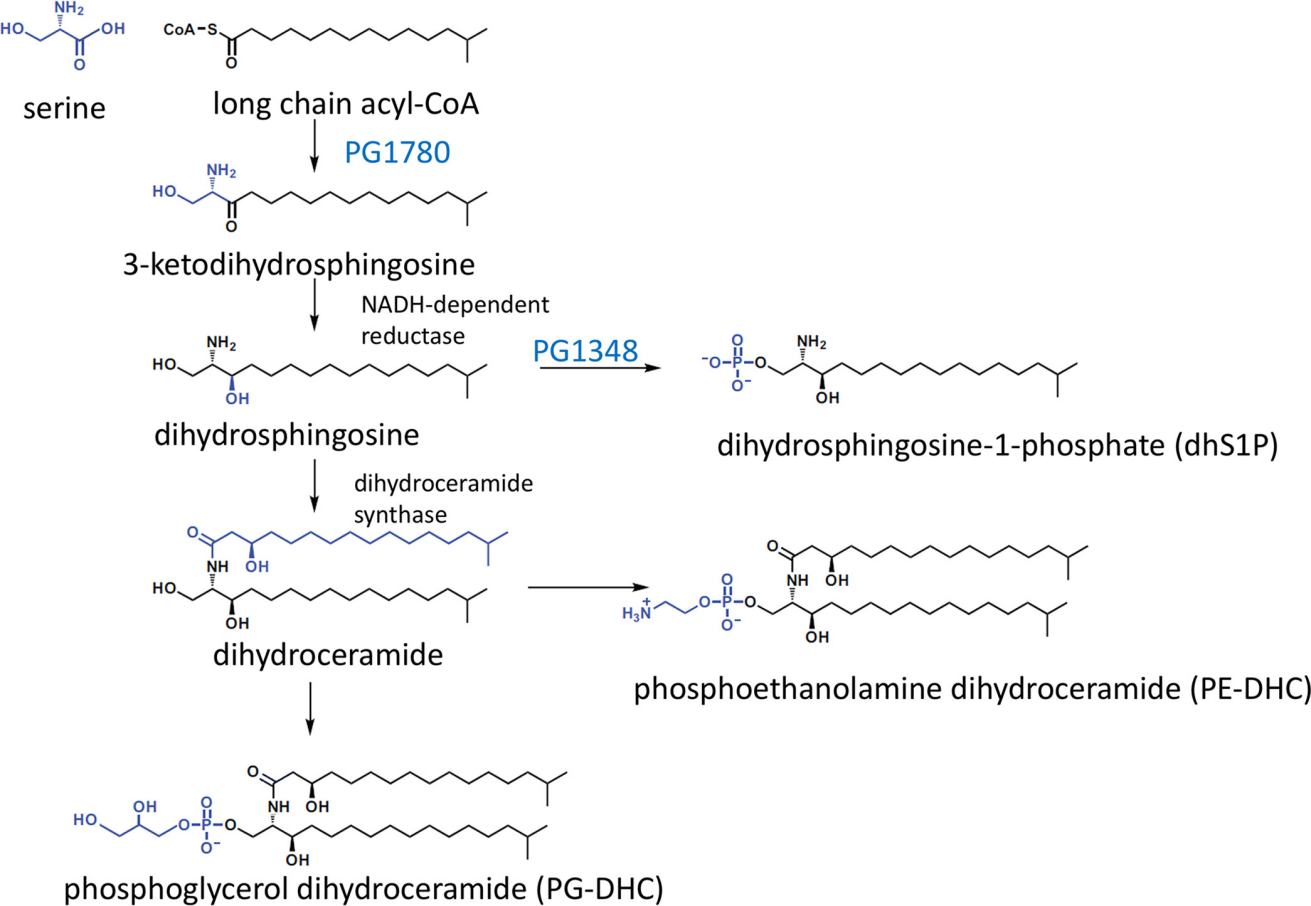
Predicted biosynthetic pathway for sphingolipid synthesis in P. gingivalis.

In this study, we identified PG1348 as the first bacterial dihydrosphingosine kinase (dhSphK1), which phosphorylates dihydrosphingosine to form dihydrosphingosine-1-phosphate (dhS1P) (Fig. 1). Our working hypothesis was that the synthesis of dhS1P diverts the flow of the dihydrosphingosine core away from the synthesis of DHCs, a model proposed in an earlier study by Wieland Brown et al. (24). Indeed, lipid analysis discovered that a Δ*dhsphK* mutant has a shift in lipid profile, producing higher levels of PG-DHCs and lower levels of PE-DHCs than the parent strain. The ΔPG1348 mutant also demonstrated a defect in K antigen capsule, suggesting a link between sphingolipid synthesis and the production of K antigen capsule. Overall, the results presented in this report show that the PG1348 gene encodes a novel dihydrosphingosine kinase whose activity influences membrane integrity, outer membrane vesicle (OMV) biogenesis, the presentation of K antigen capsular polysaccharide, survival, and virulence.

## RESULTS

### P. gingivalis PG1348 structurally resembles the mammalian sphingosine kinase SphK1.

**FIG 2:**
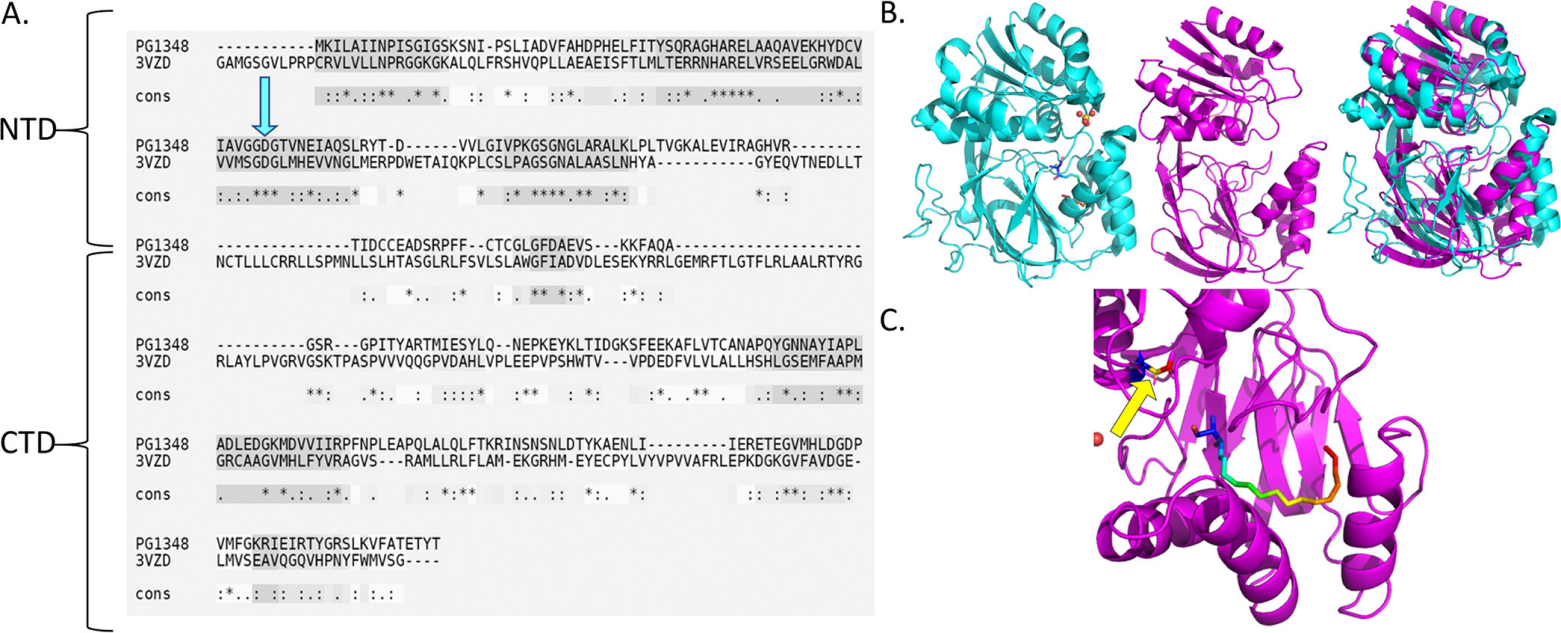
Bioinformatics and computational analysis of PG1348 (dhSphK1). The amino acid sequence of PG1348 was analyzed using I-TASSER. The closest match was the human sphingosine kinase SphK1 (PDB code 3VZB). (A) Sequence alignment of PG1348 and SphK1 (3VZB) using T-COFFEE Expresso web-based software. Blue arrow indicates conserved catalytic center residue Asp81 in SphK1, corresponding to Asp66 in PG1348. (B) Comparison of three-dimensional structure of SphK1 (3VZB) in cyan with predicted structure of PG1348 in magenta and their superimposition. (C) Predicted catalytic center of dhSphK1, showing a rainbow-colored stick structure that denotes sphingosine in a space-fitting model with different colors indicating different side chains. Yellow arrow indicates Asp66.

In a previous search for the biosynthetic pathway of sphingolipids in P. gingivalis, we identified and characterized PG1780, a serine palmitoyltransferase (SPT). As shown in Fig. 1, this enzyme catalyzes the first committed step in sphingolipid biosynthesis, the condensation of serine and long-chain acyl-CoA (22, 26). The genome-wide search for other sphingolipid-biosynthetic genes identified the gene encoding PG1348, annotated as a sphingosine kinase gene. Importantly, BLASTP mapping across 60 P. gingivalis strains found that, in 11 strains, the gene is identical. In 47 strains, there is a single mismatch in the full sequence, and the last 2 strains have two mismatches; therefore, the PG1348 gene is well conserved in all strains. To characterize a possible structural and functional role for PG1348, we analyzed the amino acid sequence for alignment and structural modeling with those of closely related proteins. Using I-TASSER (27, 28), we determined that the predicted structure of PG1348 aligned most closely (highest TM-align score) with the human sphingosine kinase SphK1 (Protein Data Bank code 3VZB), which is a well-studied enzyme that converts sphingosine to sphingosine-1-phosphate (Fig. 2A and B). According to this model, PG1348 harbors a 2-domain architecture with an N-terminal ATP binding domain (NTD) and a C-terminal sphingosine binding domain (CTD), similar to SphK1 (Fig. 2B). The catalytic center of mammalian SphK1 is located between the two domains, and the position of Asp81, which was assigned as the general base responsible for the deprotonation of sphingosine, is also conserved in PG1348 as Asp66 (Fig. 2A and C, blue arrow and yellow arrow, respectively). Unlike other lipid kinases, SphK1 is characterized by a catalytic center that makes extensive surface contact with the sphingosine substrate, and access of the substrate to the catalytic center is likely mediated by the opening and closure of the lipid binding loop, as shown in Fig. 2C (29). Given the structural similarity with SphK1, PG1348 was annotated as a sphingosine kinase capable of converting sphingosine to sphingosine-1-phosphate; however, as shown below, our studies determined that the preferred substrate is actually dihydrosphingosine, the fully saturated form of sphingosine, also known as sphinganine.

### PG1348 is not a sphingosine kinase, instead it phosphorylates dihydrosphingosine.

**FIG 3:**
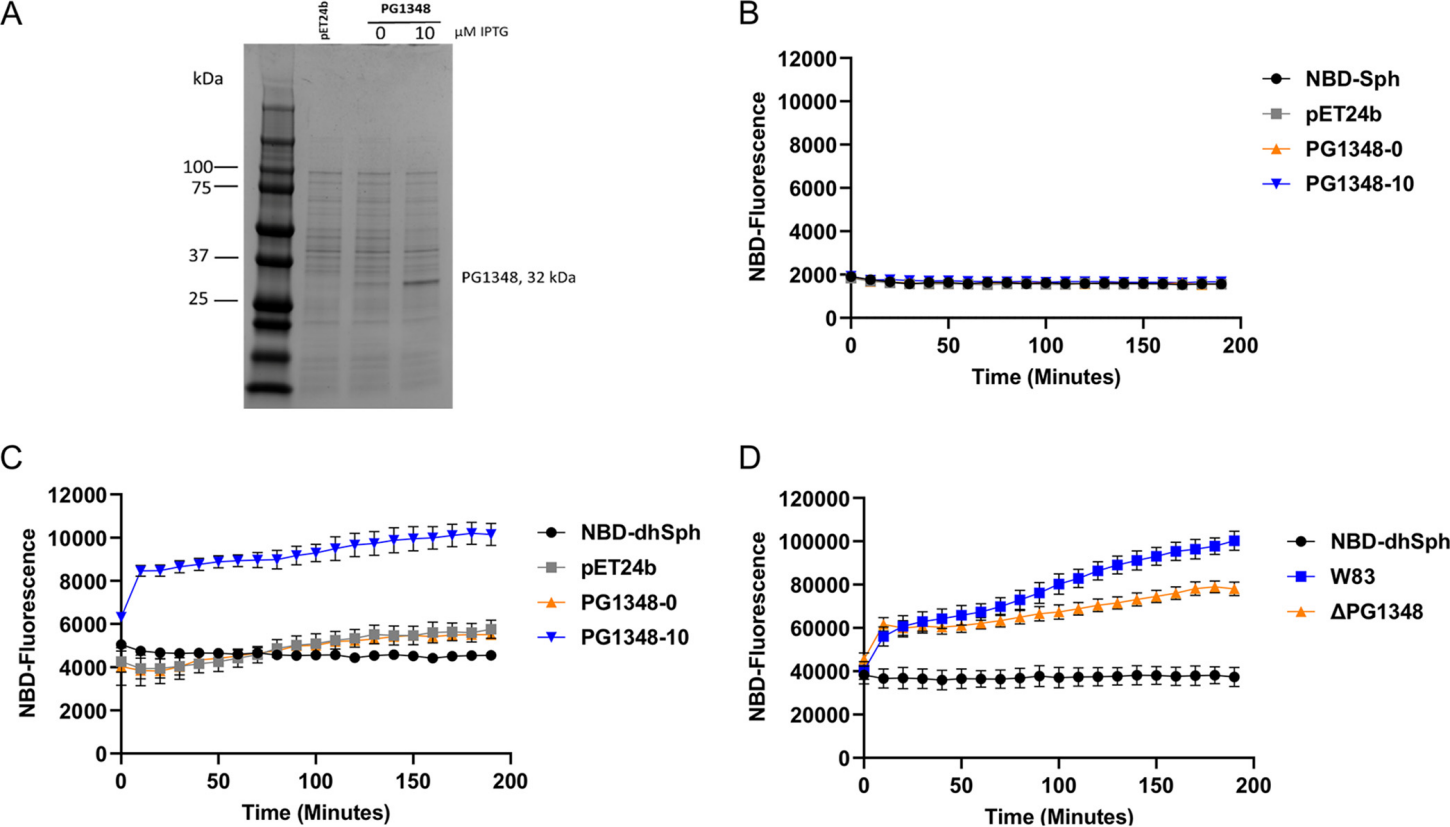
Fluorescence-based sphingolipid kinase assay using NBD-labeled lipid substrates. (A) Expression of PG1348 from pET24b in E. coli analyzed by protein gel electrophoresis. Lanes: 1st, protein ladder; 2nd, empty vector pET24B; 3rd and 4th, pET24b-PG1348 uninduced and induced with 10 μM IPTG, respectively. (B) NBD-sphingosine assay on cell lysates from E. coli. (C) NBD-dihydrosphingosine assay on cell lysates from E. coli. (D) NBD-dihydrosphingosine assay on cell lysates from the parent strain W83 and the W83 ΔPG1348 mutant. Number of repeats (*n*), 3. Error bars shown in the graph were calculated using the following formula: SE = SD/√*n*, where SE is the standard error of the sample, SD is the standard deviation of the sample, and *n* is the number of samples.

To determine if the PG1348 protein has sphingosine kinase activity, we employed a fluorescence-based assay that was developed for reliable and real-time detection of sphingosine kinase activity (30). This assay uses 7-nitro-2-1,3-benzoxadiazol-4-yl (NBD)-labeled sphingosine or sphinganine (dihydrosphingosine) as the substrate, emitting yellow color upon excitation at 463 nm and detecting conversion into NBD-sphingosine-1-phosphate by the spectrum shift to red light at an excitation of 550 nm (30). Although, due to solubility issues, the expression and purification of PG1348 using various constructs was unsuccessful, this fluorescence-based kinase assay could be employed because it can be applied to whole-cell lysates to examine cellular sphingosine kinase activity (31). We therefore generated a PG1348 deletion mutant in P. gingivalis strain W83, grew both the ΔPG1348::Erm mutant and the parent strain overnight, lysed the cells by sonication to generate whole-cell lysates, and examined whether PG1348 could convert NBD-sphingosine into NBD-sphingosine-1-phoshate, as described in Materials and Methods. Surprisingly, P. gingivalis failed to show any phosphorylation activity when NBD-sphingosine was provided as a substrate. We first reasoned that the level of PG1348 in the wild-type (WT) strain might not have been sufficient to be detected by the fluorescent assay. To resolve this, we generated an expression system in Escherichia coli to overproduce PG1348 using the pET24b vector and confirmed the induction of PG1348 by protein gel analysis (Fig. 3A). However, when induced E. coli lysates were examined for sphingosine kinase activity, again no fluorescence was detected with NBD-sphingosine as the substrate (Fig. 3B) showing that PG1348 did not phosphorylate sphingosine under our assay conditions.

Next, we reasoned that the structural differences between sphingosine and dihydrosphingosine (the natural substrate in P. gingivalis) could impact substrate specificity. We therefore provided NBD-dihydrosphingosine as the substrate in the fluorescence-based kinase assay. The fluorescent signals for the E. coli lysates from an induced culture (10 μM IPTG [isopropyl-β-d-thiogalactopyranoside]) displayed high levels of activity compared to the results for E. coli expressing the empty vector (pET24) and an uninduced control (0 μM IPTG [PG1348-0]). The controls generated low levels of fluorescence, similar to the level in the NBD-dihydrosphingosine-only control (Fig. 3C). These results showed that, while PG1348 could not phosphorylate sphingosine under these assay conditions, this enzyme could phosphorylate dihydrosphingosine. To confirm the presence of dihydrosphingosine kinase activity in P. gingivalis extracts, we generated lysates from the ΔPG1348 mutant and the W83 wild type. The fluorescence produced by lysates of the mutant was significantly lower than that in the parent strain, indicating that PG1348 contributes to dihydrosphingosine kinase activity (Fig. 3D). However, the ΔPG1348 mutant still showed some level of fluorescence compared to the results for the negative control (NBD-dihydrosphingosine only) (Fig. 3D), showing that P. gingivalis contained additional kinases that phosphorylated dihydrosphingosine. These results were further verified when the levels of dihydrosphingosine-1-phosphate (dhS1P) were analyzed in the parent strain and the W83 ΔPG1348 mutant cells, showing that dhS1P was still detected in the mutant (Fig. S5 in the supplemental material). Based on the substrate specificity, we propose PG1348 as a dihydrosphingosine kinase and reannotate the PG1348 gene as *dhsphK1*. The results indicate that dihydrosphingosine is the preferred substrate for dhSphK1 (protein designation) and, importantly, that this activity produces the signaling molecule dhS1P.

### dhSphK1 activity impacts the synthesis of sphingolipid subtypes in P. gingivalis.

**FIG 4:**
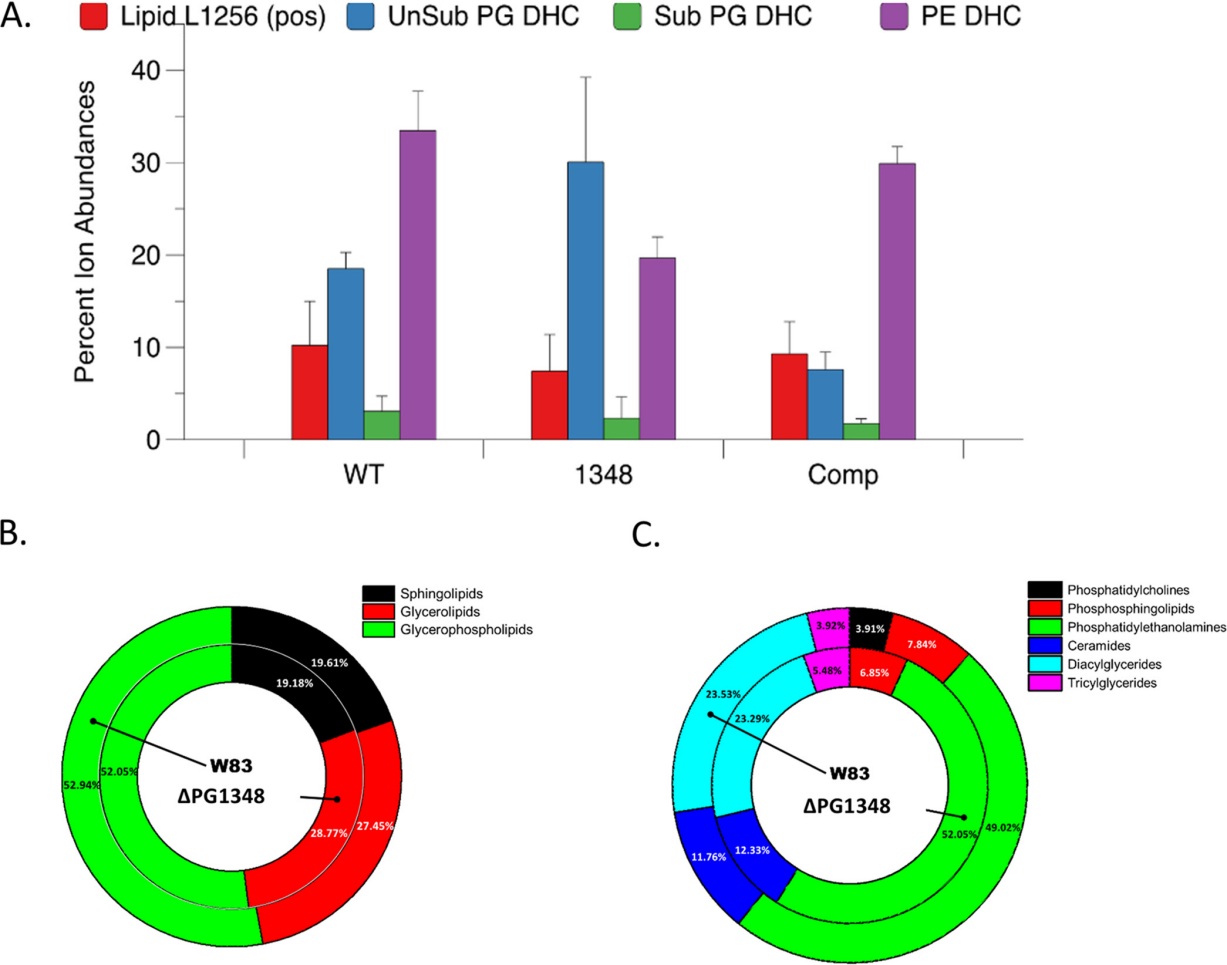
Deletion of *dhsphK1* impacts the lipid composition of P. gingivalis. (A) Total lipid extracts were analyzed for the relative levels of a serine-glycine lipid (lipid 1256) and various sphingolipid classes in the wild type (WT), the Δ*dhsphK1* mutant (1348), and the complemented strain (Comp [pTCOW-1348]). The data represent the average values from three biological repeats. Number of repeats (*n*), 3. Error bars shown in the graph were calculated using the following formula: SE = SD/√*n*, where SE is the standard error of the sample, SD is the standard deviation of the sample, and *n* is the number of samples. (B and C) Donut plots for untargeted lipidomics for the wild type (W83) and the Δ*dhsphK1* mutant showing (B) relative amounts of overall lipid classes and (C) relative amounts of overall lipid subclasses.

As noted above, we have shown that a sphingolipid-null mutant can be generated in P. gingivalis with the deletion of SPT (PG1780), which catalyzes the first committed step in sphingolipid synthesis (22). Our working model has been that synthesis of dhS1P by PG1348 may play a role in the synthesis of different sphingolipid subtypes by diverting the flow of the dihydrosphingosine core away from the sphingolipid biosynthesis pathway. To test this, we compared the sphingolipid compositions of the Δ*dhsphK1* mutant and the parent strain. Bacterial lipids were extracted from overnight broth cultures as described in Materials and Methods. Mass spectrometric (MS) analysis of cell extracts demonstrated that the PE-DHC lipid class was the most abundant dihydroceramide lipid in wild-type P. gingivalis relative to the abundances of other phosphorylated sphingolipid classes (Fig. 4A and Fig. S1). Interestingly, the Δ*dhsphK1* (PG1348 gene deletion) mutant demonstrated a marked increase in PG-DHCs, with a concomitant decrease in PE-DHC lipids (Fig. 4A), and the complemented strain, carrying *dhsphK1*, restored the relative levels of PE-DHC and PG-DHC lipids to those observed in the parent strain (Fig. 4A). These results demonstrate that *dhsphK1* can influence the ratio of PE-DHCs to PG-DHCs in P. gingivalis.

To analyze the impact of *dhsphK1* on the overall lipid profile of P. gingivalis, we performed untargeted lipidomic analysis by mass spectrometry. Interestingly, we found that extracts from P. gingivalis were composed of approximately 20% sphingolipids and 80% glycerolipids (including glycerophospholipids) in both the wild type and the Δ*dhsphK1* mutant (Fig. 4B), indicating that the total sphingolipid content of the Δ*dhsphK1* mutant was not altered. However, in the case of glycolipid content, there were notable differences observed, such as for phosphatidylcholines, which constituted about 4% in the wild type but were undetectable in the Δ*dhsphK1* mutant (Fig. 4C). Similarly, deleting *dhsphK1* altered the species of other lipids, including the levels of monoacylglycerols, glycerophosphoethanolamines, and free fatty acids (Fig. S1). These results indicate that the deletion of *dhsphK1* had a global impact on the overall lipid synthesis in P. gingivalis cells.

### Deletion of the PG1348 gene (*dhSphK1*) alters the morphology, growth, OMV biogenesis, cell surface composition, and lipid profile of P. gingivalis, yet there is no change in gingipain activity.

**FIG 5:**
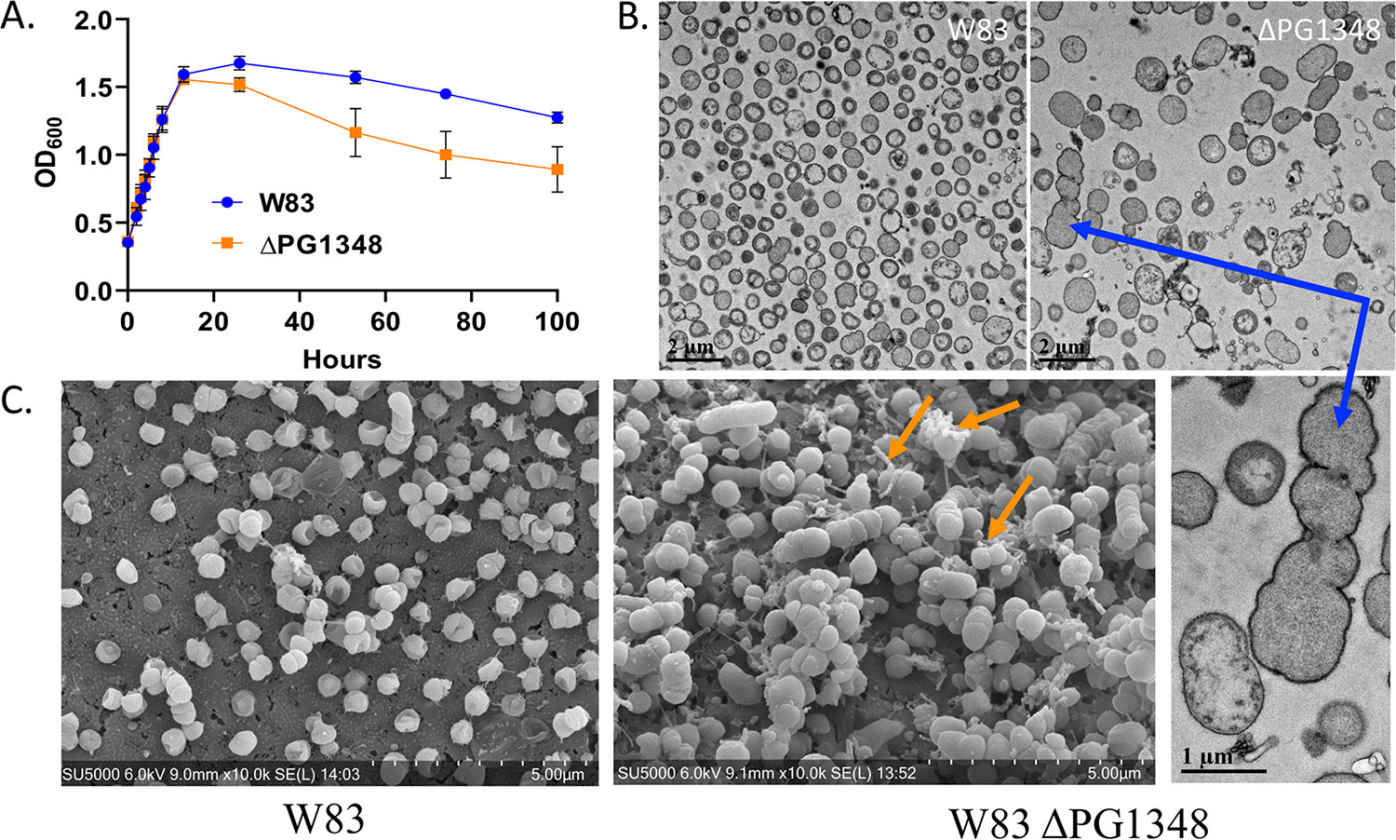
Deletion of *dhsphK1* alters the growth and cell morphology of P. gingivalis strain W83. (A) Growth curves for the wild type (W83) and the Δ*dhsphK1* mutant. Number of repeats (*n*), 3. Error bars shown in the graph were calculated using the following formula: SE = SD/√*n*, where SE is the standard error of the sample, SD is the standard deviation of the sample, and *n* is the number of samples. (B) Transmission electron micrographs of the wild type (W83) and the Δ*dhsphK1* mutant showing a cell division defect and membrane ruffling (blue arrow) in the mutant. (C) Scanning electron micrographs showing an altered OMV biogenesis phenotype (OMV chains, orange arrows) in the Δ*dhsphK1* mutant.

**FIG 6:**
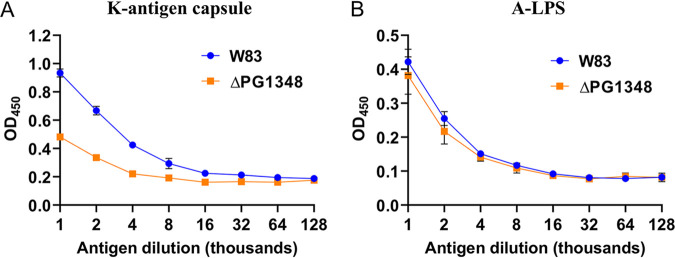
Enzyme-linked immunosorbent assays (ELISAs) to detect relative amounts of K antigen capsular polysaccharide (A) and anionic polysaccharide (B) in the wild type (W83) and the Δ*dhsphK1* mutant. Lower levels of K antigen capsule were detected in the Δ*dhsphK1* mutant than in the parent strain, and yet, the level of APS was not altered. Number of repeats (*n*), 3. Error bars shown in the graph were calculated using the following formula: SE = SD/√*n*, where SE is the standard error of the sample, SD is the standard deviation of the sample, and *n* is the number of samples.

To examine the effects of *dhsphK1*-directed shifts in the lipid profile on P. gingivalis physiology, we compared the presentation of cell surface polysaccharides and growth of the Δ*dhsphK1* mutant with those of P. gingivalis W83. When grown in broth culture, the Δ*dhsphK1* mutant grew similarly to the wild type during the exponential and early stationary phase up to 13 h. However, after 13 h, the density of W83 lacking *dhsphK1* started to decline rapidly compared to the growth curve of the parent strain, showing a significant difference at 100 h (Fig. 5A). This indicated that dhSphK1 activity influenced survival during the stationary phase. To investigate morphological changes, we examined cells grown to stationary phase using transmission electron microscopy (TEM) and scanning electron microscopy (SEM). Under TEM, the Δ*dhsphK1* strains exhibited altered cell morphology with enlarged size (Fig. 5B). These cells were elongated and showed irregular membrane ruffling, suggesting membrane damage or defects in cell division (Fig. 5B). Cellular debris and membrane fragments in the preparations from the mutant strain were also observed, suggesting active cell lysis. Furthermore, when the cells were grown to late exponential phase and examined by SEM, it was evident that the mutant had a cell division defect and produced long chains of OMVs, unlike the parent strain, which released high levels of individual OMVs. In addition, since earlier studies showed that the sphingolipid-null mutant had lower levels of K antigen capsular polysaccharide (22), we examined the impact of *dhsphK1* on polysaccharides presented on the cell surface. Enzyme-linked immunosorbent assays (ELISAs) were performed to detect K antigen capsule, as well as anionic polysaccharide (APS). The assays detected a lower level of K antigen capsule in the Δ*dhsphK1* mutant than in the parent strain, and yet, the level of APS was not altered (Fig. 6A and B). Importantly, as shown by the results in Fig. S3, there was no change in cell-associated or secreted gingipain activity in the mutant compared to that in the parent strain W83. This aligns with no defect in the levels of A-LPS, which is known to anchor the gingipains to the cell surface. Together, these results indicate that dhSphK1 activity influences membrane integrity, OMV biogenesis, the presentation of K antigen capsular polysaccharide, and survival during stationary phase.

### Deletion of the PG1348 gene (*dhsphK1*) impacts the transcriptome of P. gingivalis.

**FIG 7:**
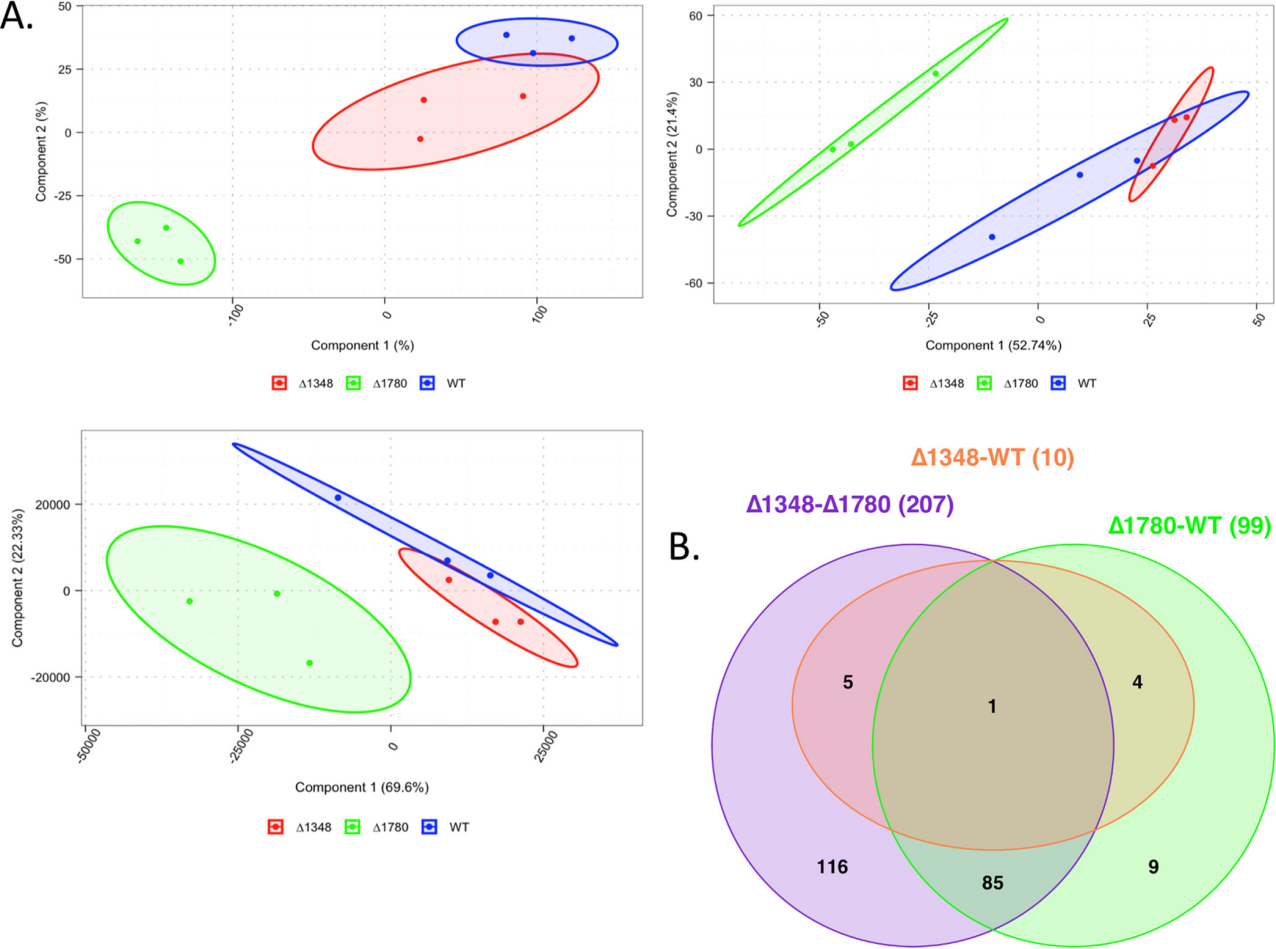
RNA-Seq overview. (A) Cluster analysis (left, *t*-distributed stochastic neighbor embedding [t-SNE]; middle, principal-component analysis [PCA]; right, principal-coordinate analysis [PCoA]) of normalized counts consistently demonstrated a separation between W83 ΔPG1780 and both W83 ΔPG1348 and the parent strain W83. This separation was not as strong for the ΔPG1348 mutant and the parent strain, which resulted in an effectively low number of differentially expressed genes in the analysis. (B) Venn diagram shows counts of differentially expressed genes across all three comparisons. As shown in the cluster, comparisons between the ΔPG1348 mutant and the ΔPG1780 mutant and between the ΔPG1780 mutant and the WT yielded 207 and 99 differentially expressed genes, respectively, while only 10 differentially expressed genes were found in the comparison of the ΔPG1348 mutant and the WT.

To analyze the global impact of *dhsphK1* on transcription, we extracted RNA from cultures grown to late exponential phase and compared the transcriptome of the Δ*dhsphK1* mutant to that of the parent strain W83, as well as the W83 ΔPG1780 sphingolipid-null strain. As shown by the results in Fig. 7, the analysis determined that the transcriptome of the Δ*dhsphK1* mutant was more similar to that of the parent strain, with only 10 genes being differentially expressed, than to the transcriptome of the W83 ΔPG1780 sphingolipid-null strain, as 207 genes were found to be differentially expressed in the Δ*dhsphK1* mutant compared to their expression in the W83 ΔPG1780 sphingolipid-null strain. Importantly, of the 10 genes that were differentially expressed in the Δ*dhsphK1* mutant (Table S2), 5 (PG0985, PG0986, PG0987, PG0050, and PG0555 genes) were also found to be differentially expressed in the ΔPG1780 mutant compared to their expression in the wild type: specifically, the operon (PG0985, PG0986, and PG0987 genes) and the gene encoding a predicted transposase (PG0050 gene) were expressed at lower levels in the mutant, while the expression of the gene encoding a histone-like protein (PG0555 gene) was detected at a higher level. This gene encoding the histone-like protein was the one gene that was differentially expressed in all comparisons. Of note, the PG0985 gene encodes the extracytoplasmic stress response sigma factor (sigma E), suggesting a common change in a stress response regulatory pathway in both mutants. In addition, when the Δ*dhsphK1* mutant transcript profile was directly compared to that of the ΔPG1780 sphingolipid-null strain (Table S3), the expression of genes in the K antigen locus (PG0106 to PG0119 genes) was detected at higher levels (>2-fold) in the Δ*dhsphK1* mutant, a change in expression that was not detected when the ΔPG1780 mutant was compared to the wild type. This finding suggested that the Δ*dhsphK1* mutant might have been trying to synthesize more K antigen capsule and/or LPS to compensate for the change in sphingolipid synthesis. Finally, approximately half of the differentially expressed genes (57 genes) encoded hypothetical proteins or proteins of unknown function.

### Deletion of PG1348 correlates with an altered inflammatory response from THP-1 cells.

**FIG 8:**
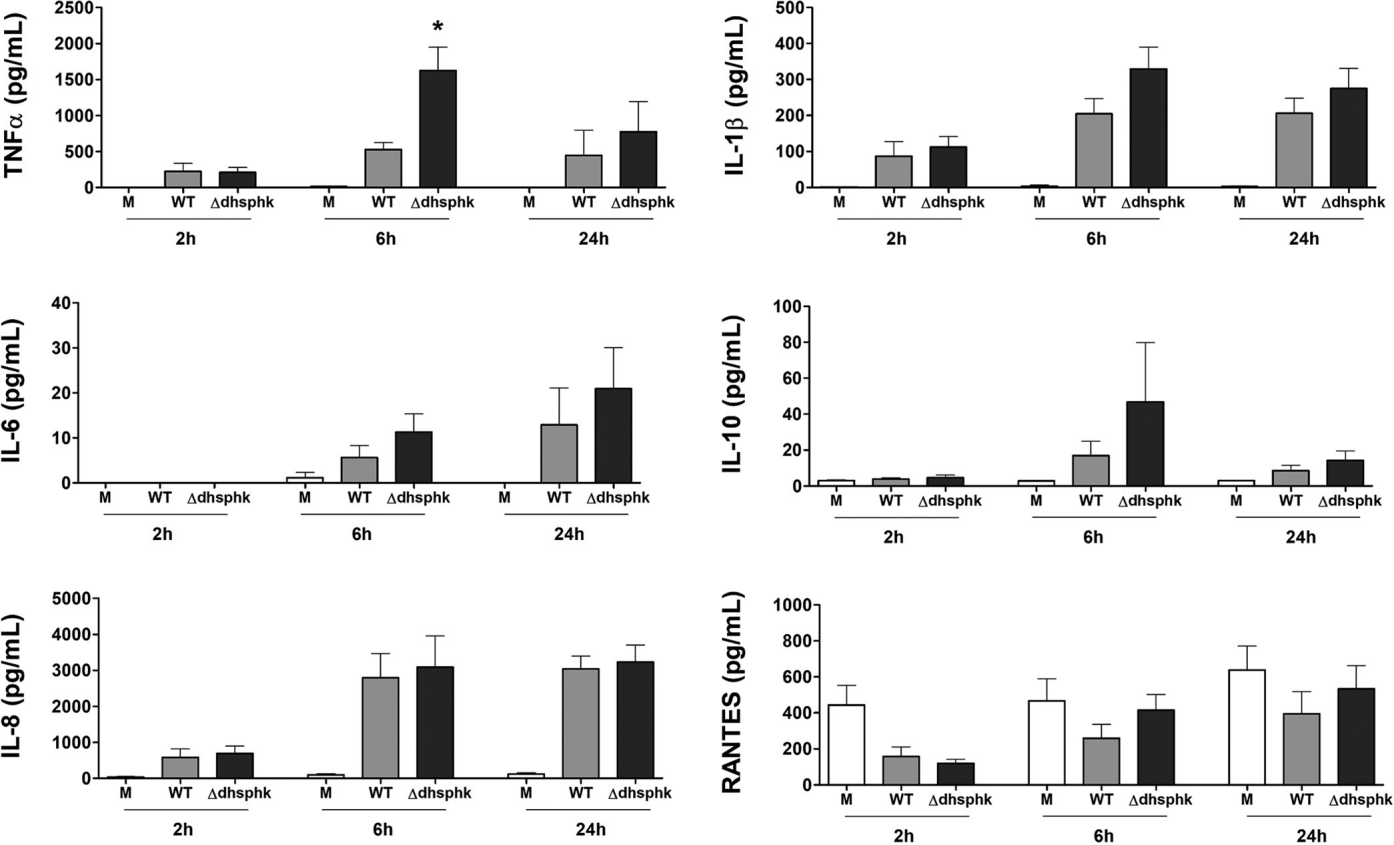
Impact of the deletion of PG1348 (dhSphK1) on cytokine and chemokine response. Human macrophage-like THP-1 cells were directly cultured with P. gingivalis W83 (WT) and the ΔPG1348 (dhSphk1) mutant cells. Supernatant fluids were collected at 2, 6, and 24 h, and the levels of TNF-a, IL-1b, IL-6, IL-10, RANTES, and IL-8 were measured by multiplex immunoassay. Medium alone (M) served as an unchallenged (negative) control. THP-1 cells cultured with the Δ*dhsphK1* mutant and with the WT produced similar level of cytokines and chemokines; however, there was a trend toward a slightly higher cytokine response to the Δ*dhsphK1* mutant. Multiplicity of infection (MOI), 100; data are presented as mean ± SEM (*n* = 3 independent experiments); *P* < 0.05.

We anticipated that altering the pool of sphingolipid subtypes and the amount of K antigen capsule would impact the inflammatory response of host cells to P. gingivalis. To test this hypothesis, we first investigated the response elicited by direct cell-to-cell contact with P. gingivalis wild type (WT) or the Δ*dhsphK1* mutant on human macrophage-like THP-1 cells and measured cytokine and chemokine levels in cell culture supernatants collected at 2, 6, and 24 h. THP-1 cells cultured with the Δ*dhsphK1* mutant and the WT produced similar level of cytokines and chemokines; however, as shown by the results in Fig. 8, there was a trend toward a slightly higher cytokine response to the Δ*dhsphK1* mutant. We have shown that sphingolipids from P. gingivalis can be delivered to the host cells by outer membrane vesicles (OMVs) and that purified OMVs from the ΔPG1780 sphingolipid-null strain induce a hyperinflammatory response from the THP-1 cells (26, 32). To explore the impact of deletion of PG1348 on OMV function, we purified OMVs from the parent strain, the ΔPG1780 mutant, and the Δ*dhsphK1* mutant and examined their effects on THP-1 cells. As reported in our previous publication (32), when THP-1 cells are exposed to OMVs from the sphingolipid-null mutant, this elicits a significantly elevated inflammatory response compared to treatment with OMVs from the WT, and importantly, this same hyperinflammatory response is elicited from direct cell-cell contact with the ΔPG1780 mutant (26). Strikingly, we observed that the levels of cytokines and chemokines secreted by THP-1 cells stimulated with OMVs from the Δ*dhsphK1* mutant were comparable to or slightly lower than the levels elicited by OMVs from the WT, especially at the 6-h and 24-h time points (Fig. S2). And yet, surprisingly, as noted above, this was not the response elicited from direct cell-cell contact, suggesting that the impact of dhSphK1 activity differentially affected P. gingivalis cells and the OMVs produced from those cells. Taken together, these findings indicated that changes in sphingolipid synthesis that resulted from the deletion of PG1348 correlated with an altered inflammatory response from THP-1 cells; further investigation will be required to determine the underlying mechanisms.

## DISCUSSION

The results presented in this report show that P. gingivalis can modulate the ratio of different sphingolipid subtypes via phosphorylation of dihydrosphingosine, a core intermediate of sphingolipid synthesis, and that this phosphorylation is, in part, controlled by a novel lipid kinase (dhSphK1 [PG1348]). The data also demonstrate that a shift to a higher level of PG-DHCs can lead to a modest response of macrophages to OMVs isolated from the P. gingivalis strain W83 Δ*dhsphK1* mutant, similar to treatment with OMVs isolated from the parent strain W83, while a sphingolipid-null strain elicits a robust inflammatory response. These findings align with our previous studies showing that when P. gingivalis synthesizes sphingolipids, there is a limited inflammatory response from THP-1 cells (26, 32).

The key enzymes involved in eukaryotic sphingolipid metabolism are two sphingosine kinases (SphK1 and SphK2) that catalyze the conversion of sphingosine to form sphingosine-1-phosphate (S1P) (33, 34). These kinases can also phosphorylate dihydrosphingosine to form dhS1P, although sphingosine is the preferred substrate (35, 36). Mammalian sphingosine kinases have been studied extensively due to their role in the production of the signaling molecule S1P, which has profound impacts on cell proliferation and differentiation, autophagy, and apoptosis, including pathophysiological roles in inflammation, autoimmune dysfunction, cancer, and many other diseases (37). Mammalian ceramide kinases that phosphorylate ceramide to produce ceramide-1-phosphate demonstrate a high specificity for substrate recognition; they do not phosphorylate sphingosine or other diacylglycerols (38, 39). Similarly, when sphingosine kinase activity was examined in embryonic fibroblasts from a sphingosine kinase knockout (*sphk1^−/−^*) mouse, the presence of other lipid kinases did not promote the conversion of sphingosine to S1P (31), suggesting that these lipid kinases can have high specificity. Based on the sequence similarity and prediction of the 3-dimensional structure of the P. gingivalis dhSphK1 compared to human SphK1, we predicted that P. gingivalis dhSphK1 would also recognize and phosphorylate sphingosine. However, the P. gingivalis dhSphK1 failed to phosphorylate sphingosine; instead, dihydrosphingosine was the preferred substrate. Since the only difference between these two molecules is the presence of the double bond on the sphingoid base structure, it appears that the specificity is determined by the double bond. In the mammalian sphingolipid classes, the double bonds that distinguish dihydroceramides from ceramides markedly alter the biophysical properties of the molecules, regulating their dipole potential and modifying the elastic properties and packing behavior (40). Although long considered biologically inactive, studies are showing that dihydroceramides can have significant effects on biological processes like autophagy, oxidative stress, cell proliferation, apoptosis, and other metabolic disorders (41, 42). Therefore, we predict that future studies on bacterially derived dihydroceramides may provide more evidence of their impact on human health. It is important to note that the regulatory mechanisms that control *dhsphK1* expression have yet to be determined and PG1348 does not appear to be the only dihydrosphingosine kinase produced by P. gingivalis. BLAST analysis found no evidence of any functional homologue to the PG1348 gene across the genome, so whether there are additional enzymes capable of phosphorylating dihydrosphingosine is still not clear.

As described above, sphingoid base phosphates like S1P and dhS1P have multiple effects on various components of the cellular immune response (43). The dynamic balance between the phosphorylated and nonphosphorylated states of S1P with free ceramide has been proposed to form a “cellular rheostat” that determines cell survival versus cell death (44). Importantly, S1P gradients and S1P receptors (G protein-coupled receptors) also play a central role in mediating immune cell migration to sites of inflammation (45). Like S1P, dhS1P also functions as a ligand for S1P receptors, and yet, the binding affinity for the different S1P receptor isoforms differs from that of S1P (46). It has been proposed that a shift in ratio to higher levels of dhS1P may elicit different downstream signaling cascades due to differential binding and activation of the various S1P receptor isoforms (46). Furthermore, high concentrations of these ligands have been shown to cause internalization and downregulation of S1P receptors, thereby acting as functional antagonists rather than as agonists (45). Hence, current research is providing additional support to the importance of exploring the role of dhS1P on human health, and therefore, the ability of P. gingivalis, as well as other members of the *Bacteroidetes*, to synthesize and potentially alter the levels of dhS1P *in vivo* is intriguing.

Ever since the major classes of sphingolipids produced by P. gingivalis (PG-DHCs, PE-DHCs, and DHCs) were identified (2), research efforts have focused on purified extracts and the role of these lipids as virulence factors. Interestingly, purified phosphorylated dihydroceramides function as Toll-like receptor ligands and may play a role in autoimmune diseases (47). Further investigations have shown that purified PG-DHCs and PE-DHCs can engage Toll-like receptor 2, promoting cell activation of dendritic cells, macrophages, or osteoblasts (47, 48), which at first glance appears incongruent with the results presented in this study, where the synthesis of DHCs appears to prevent activation. Surprisingly, when gingival tissue samples were examined for the relative levels of PG-DHCs and PE-DHCs, bacterial sphingolipids were recovered from both healthy and diseased gingival tissues. However, the study also showed that a higher ratio of PG-DHCs to PE-DHCs was recovered from the healthy gingival tissues (3); therefore, like dhS1P and S1P, the ratio of the different types of bacterial DHCs may constitute a key signal. Our working hypothesis is that a higher ratio of bacterially derived PG-DHCs to PE-DHCs is associated with homeostasis and potentially an indicator of health.

In these studies, we also show that sphingolipids comprise ∼20% of total cellular lipids and that this proportion remains unaltered in the Δ*dhsphK1* mutant, suggesting that the phosphorylated products of dhSphK1 activity are retained or recycled in cellular lipid pools. The composition of sphingolipid subtypes showing an increased level of PG-DHC when dhSphK1 activity is absent suggests a possible feedback inhibition of PG-DHC synthesis by dhS1P in P. gingivalis. The results also show that the Δ*dhsphK1* mutant is altered in cell morphology, showing enlarged, elongated cells with irregular membrane ruffling, indicating membrane damage and defects in cell division. Cell debris and membrane fragments are also observed, suggesting cell lysis, which aligns with the observed decrease in survival during late stationary phase, similar to the findings for a previously characterized ΔPG1780 (serine palmitoyltransferase) mutant, which is unable to synthesize any DHCs. It is possible that sphingolipids play a role by positioning protein assemblies in the membrane for synthesis of K antigen capsule and/or that a subset of DHCs may play a more direct role in anchoring capsule to the outer membrane. Furthermore, since sphingolipids are known to generate favorable microdomains that stabilize select membrane proteins involved in signaling, shifts in the types of DHCs synthesized may also alter signaling pathways.

**FIG 9:**
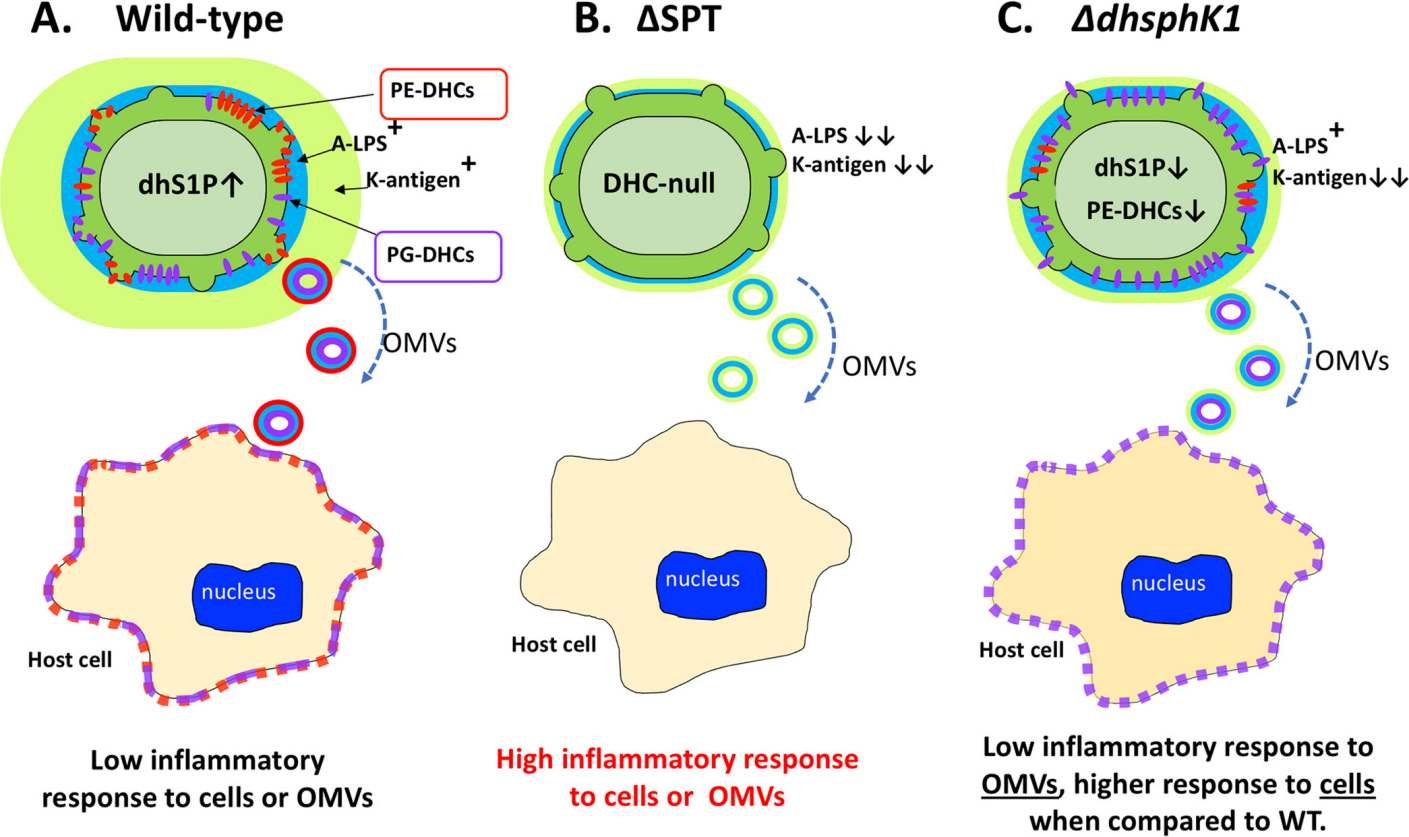
Working model showing the link between synthesis of dhS1P and sphingolipids (PE-DHCs and PG-DHCs) and the ability of P. gingivalis to elicit an inflammatory response in THP-1 cells. (A) Our studies have shown that sphingolipids produced by the wild-type strain W83 are delivered to host cells (THP-1) via outer membrane vesicles (OMVs) or cell-to-cell contact and that this correlates with a low inflammatory response to W83. (B) In contrast, the W83 ΔSPT mutant, which is unable to synthesize any of the sphingolipids or dihydrophingosine-1-phosphate (dhS1P), elicits a robust inflammatory response to both the OMVs and cells. (C) Higher levels of PG-DHCs in relation to PE-DHCs produced by the Δ*dhsphK1* mutant correlate with a high level of tolerance (elicits a low inflammatory response) to its OMVs, and yet, surprisingly there is a slightly higher response to Δ*dhsphK1* cells than to the parent strain. We posit that dhS1P (which is anti-inflammatory) is contained within the cytoplasm of P. gingivalis and that this signaling molecule is released upon lysis or is transported out of the cell, and hence, there is a stronger inflammatory response to the Δ*dhsphK1* mutant cells than to the parent strain. 75- by 2.1-mm ACE Excel 2 C_18_ column heated.

As noted above, the detection of a higher ratio of PG-DHCs to PE-DHCs in the Δ*dhsphK1* mutant mimics the recovery of sphingolipids from gingival tissue of healthy individuals (3). Furthermore, metatranscriptomic analysis of human subgingival plaque has shown that *dhsphK1* (the PG1348 gene, or PGN_1137) is elevated in gingival tissues that progress from gingivitis to periodontitis (49, 50), which aligns with *in vivo* results showing a shift to a higher ratio of PE-DHC in diseased periodontal tissues. Finally, the reduced inflammatory response observed in our interrogation of THP-1 cells using purified OMVs from the Δ*dhsphK1* mutant suggests that P. gingivalis can modulate the immune response through a dhSphK1-mediated change in the ratio of PG-DHCs to PE-DHCs (higher levels of PG-DHCs being more suppressive). In contrast, when the Δ*dhsphK1* cells (instead of the OMVs) were compared to the WT cells, the Δ*dhsphK1* cells were more inflammatory, not less. As shown in Fig. 9, we propose that dhS1P, which is anti-inflammatory, is primarily a cell-associated molecule, and since the mutant cells are generating less dhS1P in our cell culture system, they elicit a stronger inflammatory response; however, further work is required to elucidate the underlying mechanism. All in all, these results suggest that there are multiple mechanisms involved in immune evasion and/or suppression that are controlled via regulation of sphingolipid synthesis by P. gingivalis.

In summary, we have identified the first bacterial dihydrosphingosine kinase, which phosphorylates dihydrosphingosine to form dhS1P. Our working model is that the synthesis of dhS1P diverts the flow of the dihydrosphingosine substrate away from the production of DHCs, which in turn alters the synthesis (or recycling) of PG-DHCs and PE-DHCs, favoring higher levels of PG-DHCs. The results also show that P. gingivalis cells contain dhS1P, which is predicted to be anti-inflammatory, and that the relative amount of K antigen capsule is reduced in the Δ*dhsphK1* mutant, while the level of APS, another key surface glycan, is not altered. Overall, this report provides further evidence that the ability of P. gingivalis to regulate the synthesis of sphingolipids is critical to its basic physiology and its ability to minimize its virulence.

## MATERIALS AND METHODS

### Bacterial strains and growth media and conditions.

P. gingivalis strain W83 and derivatives were routinely grown on agar plates containing Trypticase soy broth supplemented with 5 μg/mL hemin, 1 μg/mL menadione (TSBHK), and 5% defibrinated sheep blood (Northeast Laboratory Services, Winslow, ME, USA) and incubated at 35°C in an anaerobic chamber (Coy Laboratory Products, Grass Lake, MI, USA) with an atmosphere containing 5% hydrogen, 10% carbon dioxide, and 85% nitrogen. To monitor growth, cultures of P. gingivalis strain W83 and derivatives were inoculated into TSBHK, grown for 24 h, and then diluted into fresh TSBHK medium. After approximately 12 h of growth (optical density at 600 nm [OD_600_] of ∼0.3 to 0.4), cultures were normalized with prereduced TSBHK medium to the same optical density (OD_600_ of 0.35). The optical density of cultures was routinely recorded throughout the exponential phase and then every 24 h once the cultures entered stationary phase.

### DNA manipulation and plasmid construction.

To construct a mutant with a deletion of the PG1348 gene, a mutagenic DNA fragment with an erythromycin resistance gene, *ermF*, was generated using the NEBuilder HiFi DNA assembly cloning kit (New England BioLabs, Ipswich, MA, USA) according to the instructions provided by the manufacturer. Briefly, primers (Table S1) were designed to generate upstream and downstream products of approximately 1 kb flanking the PG1348 gene, as well as the resistance gene *ermF*, obtained from plasmid pVA2198 (51). Primers were designed to incorporate sufficient overlapping regions at the ends of the products to permit the assembly of the erythromycin gene with the flanking regions of the PG1348 gene. Products were generated using Phusion high-fidelity PCR master mix with HF buffer (New England BioLabs, Ipswich, MA, USA), and equal portions of these products were mixed and incubated with the NEBuilder HiFi DNA assembly cloning kit according to the manufacturer’s instructions. The product generated using the assembly cloning kit was amplified by PCR (using the Phusion high-fidelity PCR master mix) and used to transform P. gingivalis W83 by electroporation. Colonies grown on the blood agar plate containing erythromycin were screened using colony PCR confirmation. The deletion of the PG1348 gene was confirmed by sequencing the genomic regions flanking the PG1348 gene. For complementation, the promoter segment of *groES* (PG0521 gene) containing 290 bp upstream from PG0521 was fused with the PG1348 coding region and cloned into the plasmid pTCOW (52), which was then transformed into W83 ΔPG1348 by electroporation. The presence of pTCOW-1348 in the ΔPG1348 mutant was maintained by supplementing the medium with 1 μg mL^−1^ tetracycline. Finally, to construct pET24b-PG1348, the coding region of the PG1348 gene was cloned into the NdeI and EcoRI sites of pET24b.

### NBD-sphingosine/-dihydrosphingosine assay.

The procedures for the fluorescence-based kinase assay were described in depth by Lima et al. (30) and adopted here. Briefly, 3 mL of overnight-grown bacterial culture was pelleted, washed with phosphate-buffered saline (PBS), and resuspended in 1 mL of lysis buffer containing 50 mM Tris/HCl buffer (pH 7.4), 150 mM NaCl, 10% (wt/vol) glycerol, 0.05% (wt/vol) Triton X-100, 1 mM dithiothreitol, 2 mM Na_3_VO_4_, 10 mM NaF, 10 mM β-glycerophosphate, 1 mM EDTA, 1× Halt protease, and phosphatase inhibitor (Thermo Fisher). The suspension was sonicated to lyse the cells and recentrifuged at 15,000 × *g* for 5 min to remove unbroken cells. Protein content in the clarified cell lysate was determined by using the bicinchoninic acid (BCA) assay (Thermo Fisher). Stock solutions of 2.1 mM NBD-sphingosine (Avanti Lipids) and NBD-sphinganine (dihydrosphingosine) (Avanti Lipids) were solubilized in 5% Triton X-100 and stored frozen at −20°C until use. Assays were performed in 100-μl reaction mixtures containing 50 μl of cell lysates and an equal amount of reaction buffer (100 mM Tris-HCl, pH 7.4, 150 mM NaCl, 1 mM Na_3_VO_4_, 10 mM NaF) containing 500 μM 4-deoxypyridoxine, 20 mM Mg-ATP, and 25 μM NDB-labeled substrate (either sphingosine or dihydrosphingosine). Fluorescence from the reaction mixture was measured using excitation at 550 nm and emission at 584 nm in Synergy H1 (BioTek, Inc.) in a 96-well microtiter plate.

### Lipid extraction and MS analysis for DHCs and dhS1P.

Bacterial cultures were grown, normalized, and extracted as described previously (22). The experiment was repeated at least five times. In brief, P. gingivalis strain W83 and derivatives were inoculated into TSBHK, grown for 24 h, and then diluted into fresh TSBHK. Once the cultures reached exponential phase, they were normalized to an OD_600_ of 1.0 and 1 mL of each culture was removed from the anaerobic chamber and centrifuged. The pellets were dissolved in chloroform/methanol/water (1.33:2.67:1, vol/vol/vol, 4 mL) as described previously (2). The mixture was vortexed at 15-min intervals for 2 h and then supplemented with 0.75 mL of chloroform and 0.75 mL of a buffer comprised of 2 N KCl and 0.5 N K_2_HPO_4_. The mixture was vortexed and centrifuged (2,000 × *g*) at 20°C for 4 h. The lower organic phase was removed and dried under nitrogen. Lipid samples were dissolved in neutral high-performance liquid chromatography (HPLC) solvent (hexane/isopropanol/water, 6:8:0.75, vol/vol/vol), the samples were centrifuged at 2,500 × *g* for 10 min, and the supernatants were removed for HPLC-mass spectrometric (MS) analysis. The bacterial lipid samples were injected over a normal-phase column (Ascentis Si, 3 cm_2.1 mm, 5 mm; Supelco Analytical) interfaced with an API 4000 QTRAP instrument (Sciex). Neutral HPLC solvent was delivered under isocratic conditions with a Shimadzu LC-10AD VP pump at a flow rate of 100 to 120 μl/min. Total-ion chromatograms were acquired using negative-ion mode and a mass range of 100 to 1,800 atomic mass units, and tandem-MS (MS/MS) acquisitions used parameters optimized for specific lipid products. The collision energies for negative-ion products were typically between −30 and −55 V, depending on the precursor ion under investigation. Negative-ion electrospray ionization (ESI) was carried out at −4,500 V, with a declustering potential of −90 V, focusing potential of −350 V, and entrance potential of −10 V. The ion source temperature was maintained at 300°C. The multiple-reaction-monitoring (MRM) negative-ion transitions for the serine dipeptide lipids of P. gingivalis, lipid 654 and lipid 430, were *m/z* 653.5/381.4 and *m/z* 430.3/382.3, respectively. Low-mass (LM) and high-mass (HM) phosphoethanolamine dihydroceramides (PE-DHCs) were monitored by *m/z* 677.5/240.0 and *m/z* 706.2/140.0 ion transitions, respectively. LM and HM unsubstituted PG-DHC (unsubPG-DHC) lipids were monitored by *m/z* 709.0/171.4 and *m/z* 737.0/171.4 ion transitions, respectively. LM and HM substituted PG-DHCs (subPG-DHC) were monitored by *m/z* 933.0/171.4 and *m/z* 961.5/171.4 ion transitions, respectively. The MRM peaks were electronically integrated for comparison of lipid class recoveries.

Liquid chromatography-mass spectrometry (LC-MS) was used to detect dihydrosphingosine-1-phosphate (dhS1P) in P. gingivalis cells. Specifically, a Sciex ultraperformance liquid chromatography (UPLC) instrument coupled to a Sciex 500 quadrupole time of flight (QTOF) tandem mass spectrometer and a 75- by 2.1-mm ACE Excel 2 C_18_ column heated to 50°C with a sample injection volume of 5 μL on a 20-μL loop were used. The mobile phase consisted of 10 mM ammonium formate, 0.1% formic acid in 40% water/60% acetonitrile (solvent A), and 10 mM ammonium formate, 0.1% formic acid in 90% isopropyl alcohol/10% acetonitrile (solvent B) was employed for gradient elution. An initial flow of 75% solvent A was held for 1.0 min before increasing linearly to 25% solvent B at 8.5 min, after which the column was reconditioned to the initial state for another 1.5 min. The total run time was 10.0 min, with a constant flow rate of 0.4 mL/min. Lipids were extracted from lyophilized pellets of the parent strain W83 and the Δ*dhsphK1* mutant using the phospholipid extraction procedure of Bligh and Dyer as modified by Garbus (53). Bacterial samples were dissolved in chloroform/methanol/water (1.33:2.67:1, vol/vol/vol, 4 mL). The mixture was vortexed at 15-min intervals for 2 h and then supplemented with 0.75 mL of chloroform and 0.75 mL of a buffer comprised of 2N KCl and 0.5N K_2_HPO_4_. The lower organic phase was removed and dried under nitrogen. The bacterial samples were then reextracted with chloroform (2 mL) after acidifying with concentrated acetic acid. Chloroform extracts were dried under nitrogen, and each extract was dissolved in 60 μL of acidic methanol (0.1% acetic acid) and transferred to an autosampler vial. The neutral and acidic lipid extracts were analyzed separately. The detection and quantification of dihydrosphingosine-1-phosphate (dhS1P) was performed in negative ESI-MS/MS mode using Sciex, Inc., software for analyte signal optimization and ion peak integration. The parameters for the Sciex 500 QTOF TurbolonSpray were curtain gas 30, ion source gas 1 at 55, ion source II at 60, and temperature of 500°C. The declustering potential was −80 V with the collision energy set at −8V. Statistical analysis for quantification of results for all compounds was performed using the Sciex 500 software. Peaks matching the predicted mass of P. gingivalis dhS1P (19-carbon-chain-length base) were identified in the extracts of P. gingivalis W83 and the Δ*dhsphK1* mutant. This analysis revealed that both the wild-type and the Δ*dhsphK1* mutant contained a low but measurable amount of lipid product, consistent with the presence of dhS1P. Further quantitative studies are required to determine if there is a decrease in the levels of dhS1P in the mutant, but it is evident that P. gingivalis produces dhS1P and that there is an additional kinase that is able to phosphorylate dihydrosphingosine.

### Enzyme-linked immunosorbent assays (ELISAs) for surface polysaccharides.

To prepare antigen, P. gingivalis strain W83 and derivatives were inoculated into TSBHK and grown for approximately 24 h. Cultures were diluted into fresh TSBHK, grown to mid-exponential phase, and normalized to an OD_600_ of approximately 1.0. Cultures were diluted 1/10, and 10-μl aliquots of each culture were spotted on blood agar plates. After 3, 5, and 7 days, cultures were scraped off the plates and placed into cuvettes containing sterile distilled water. Cultures were normalized to an OD_600_ of 0.5. The suspension (1.5 mL) was pelleted, and half the supernatant was removed. The cells were resuspended in the final 0.75 mL and autoclaved at 120°C for 30 min. Once cooled, the autoclaved extracts were centrifuged, and the supernatants were removed and saved for analysis.

For the quantification of surface polysaccharides, ELISAs were performed as previously described (54). Briefly, autoclaved extracts of P. gingivalis strain W83 and derivatives (described above) were diluted in 50 mM carbonate/bicarbonate buffer, pH 9.6, and serially diluted in a 96-well plate; plates containing diluted antigen were incubated at 4°C overnight. After washing plates with PBS containing 0.05% Tween 20 (PBS/Tween), a solution of 5% milk powder in PBS was used to block wells for approximately 1 h at room temperature. After washing with PBS/Tween, wells were incubated for 1 h at 37°C with a serotype-specific antiserum previously generated against P. gingivalis strain W83 (55) and diluted to a concentration of 1:2,000 in PBS containing 0.1% Tween 20 and 0.1% bovine serum albumin (PTB) to detect the capsule. Next, wells were washed with PBS/Tween and then incubated for 1 h at 37°C with a goat anti-rabbit IgM-horseradish peroxidase (HRP) antibody diluted at 1:5,000 in PTB. To detect anionic polysaccharide (APS), the monoclonal antibody 1B5 (kindly provided by Michael Curtis, Queen Mary University of London, London, England) was used at a concentration of 1:100, and anti-mouse IgG-HRP at 1:5,000 was used as a secondary antibody. After a final wash with PBS/Tween, wells were incubated with 3,3′,5,5′-tetramethylbenzidine (Sigma-Aldrich, St. Louis, MO, USA) until sufficient color appeared. Then, the action was stopped with an equal portion of 1 M HCl, and the absorbance at OD_450_ was recorded.

### Gingipain assays.

The activities of arginine and lysine gingipains were assessed as previously described (56). Briefly, cultures were inoculated into TSBHK, grown for 24 h, and diluted into fresh TSBHK. Once the cultures reached exponential phase, they were normalized to and OD_600_ of 1.0, and 1 mL of each culture was removed from the anaerobic chamber and centrifuged. The supernatants of these cultures were saved, while the pellets were resuspended in assay buffer (200 mM Tris, 5 mM CaCl_2_, 150 mM NaCl, and 10 mM l-cysteine at pH 7.6). Cultures and supernatants were diluted 1/10 in assay buffer and then serially diluted in assay buffer across a 96-well microtiter plate. The initial optical density at 405 nm was recorded, and the plates were placed in a 37°C incubator for 10 min to equilibrate the temperature. *N*-α-benzoyl-l-arginine-*p*-nitroanilide (BAPNA) or *N*-α-acetyl-l-lysine-*p*-nitroanilide (ALPNA) was added to the wells at a final concentration of 1 mM, and the microtiter plates were incubated for 2 h at 37°C. The final optical density (OD_405_) of the wells was recorded, and the difference between the initial and final optical density was reported. Experiments were performed independently at least three times with similar results.

### RNA extraction, sequencing, and bioinformatics.

P. gingivalis strain W83 and the PG1348 mutant were inoculated into prereduced TSBHK, grown anaerobically to stationary phase, and subcultured into prereduced TSBHK. Cultures were grown to an OD_600_ of 1.0. RNA extraction and sequencing were performed as described previously (57). Briefly, cells were lysed using 600 μl of TRI reagent (Zymo Research). The samples were centrifuged at 14,000 × *g* for 10 min to remove cell debris, followed by mixing with equal volumes of 100% ethanol. The mixtures were transferred to a Zymo-Spin IIC column, centrifuged for 30 s at 14,000 × *g*, and washed with 400 μl of RNA wash buffer. The samples were incubated with 80 μl of DNase I (added directly to the column) at 30°C for 1 h, washed with 400 μl of RNA wash buffer, and incubated with 80 μl of DNase I a second time under the same conditions to completely remove any contaminating DNA. Finally, the columns were washed with 400 μl of Direct-zol RNA prewash buffer, followed by 700 μl of RNA wash buffer, and the RNA samples were eluted by briefly incubating the column with nuclease-free water, centrifuging in a clean microcentrifuge tube, and repeating this elution a second time to concentrate the sample. RNA samples were delivered to the gene expression and genotyping core of the Interdisciplinary Center for Biotechnology Research (ICBR) at the University of Florida for sample quality determination and sequencing. Quality control of RNA samples was performed using a Qubit 2.0 fluorometer (Thermo Fisher, Invitrogen, Grand Island, NY) to quantify the RNA, and an Agilent 2100 bioanalyzer (Agilent Technologies, Inc.) was utilized to assess the quality. Only samples with an RNA integrity number (RIN) of 7.0 or greater were retained for further analysis.

Bioinformatics was conducted on the HiperGator3 cluster computer at the University of Florida. Quality control with FASTQC (58) revealed a good quality for all the forward reads across all samples and, as expected, a more compromised quality toward the end of the reversed reads across all samples. This drop in quality was resolved with Cutadapt (59) at a quality cutoff of 30 (29) and a minimum length of 100 for both paired-end-reads files. Additionally, Cutadapt was set to remove any sequencing adapter contamination in the forward and reversed reads. Local alignment of curated sequences against the reference genome of P. gingivalis strain W83 (NCBI accession number NC_002950.2) was conducted with the BWA-MEM algorithm (60). Alignment files (SAM/BAM) were sorted with Samtools (61) and parsed for counts. Mapped reads were counted across the reference genome with htseq-counts. Finally, the matrix of counts across all samples was analyzed in R statistical language (62) (https://www.r-project.org/) on a desktop computer. Expression analysis was conducted with the R package edgeR (63) with *P* values and log_2_ fold change cutoff thresholds of <0.05 and >1, respectively. Cluster, volcano, and Venn diagrams were also locally generated in R.

### SEM and TEM.

P. gingivalis strain W83 and the PG1348 mutant were inoculated into prereduced TSBHK, grown anaerobically to stationary phase, and subcultured using TSBHK. Cultures were grown to an OD_600_ of 1.0. At this point, the samples were immediately removed from the anaerobic chamber, chilled on ice, and delivered to the electron microscopy core at the Interdisciplinary Center for Biotechnology Research (ICBR) located at the University of Florida for scanning and transmission electron microscopy analysis (SEM and TEM, respectively). For SEM, the samples were fixed with 2.5% glutaraldehyde, 4% paraformaldehyde in PBS, pH 7.24. Cells were then washed with 1× PBS, followed by deionized water, and then dehydrated in a graded ethanol series (25%, 50%, 75%, 95%, and 100%), followed by treatment with hexamethyldisilazane (HMDS). Dried samples were mounted on aluminum stubs with carbon adhesive tabs, sputter coated with Au/Pd (Denton DeskV), and imaged with a Hitachi SU5000 FE-SEM (Hitachi High Technologies, USA). For TEM analysis, the cell pellets were washed with PBS, and the cell suspensions were fixed at room temperature for 2 h with 3.6% glutaraldehyde in 0.1 M cacodylate buffer (pH 7.2), followed by secondary fixation at room temperature in 2% osmium tetroxide in 0.1 M phosphate buffer (pH 7.2) for 1 h. Ruthenium red (0.075%) and lysine (55 mM) were added to the glutaraldehyde fixative to preserve polysaccharide-containing material. The grids were viewed with an FEI Tecnai G2 spirit twin TEM (FEI Corp.), and digital images were acquired with a Gatan UltraScan 2,000- by 2,000-pixel camera and Digital Micrograph software (Gatan, Inc.).

### Cell culture, infection, and cytokine/chemokine profiling.

Cell culture and cytokine profiling were performed as described previously (26, 32). The human monocyte cell line THP-1 (ATCC, Manassas, VA) was cultured in RPMI 1640 (Corning) supplemented with 10% heat-inactivated fetal bovine serum at 37°C in a 5% CO_2_ incubator. THP-1 cells were adjusted to 5 × 10^5^ viable cells/mL and placed into fresh medium with 100 ng/mL phorbol 12-myristate 13-acetate (PMA; Sigma-Aldrich, St. Louis, MO) to induce differentiation into a macrophage-like state. After 48 h of incubation and cell washing, sterile 0.4-mm transwell inserts were placed in the wells with THP-1 cells and the inserts were filled with 125 mL of medium and P. gingivalis W83 parent strain or derivatives. After 2, 6, and 24 h, the cell culture supernatant fluids were collected and the levels of tumor necrosis factor alpha (TNF-α), interleukin-1β (IL-1β), IL-6, IL-8, IL-10, and RANTES were determined by using Milliplex multiplex assays (EMD, Millipore, Billerica, MA). Data were acquired on a Luminex 200 system running xPONENT 3.1 software (Luminex, Austin, TX) and analyzed using a 5-parameter logistic spline curve-fitting method using Milliplex Analyst version 5.1 software (Vigene Tech, Carlisle, MA).

### Purification of OMVs and interrogation of THP-1 cells.

Outer membrane vesicles (OMVs) from the wild type and mutants derived from P. gingivalis strain W83 were isolated and purified as described previously (32). Briefly, bacterial cultures were grown initially in TSBHK medium before being subcultured and grown in RPMI 1640 medium supplemented with 10% serum under anaerobic conditions at a final volume of 500 mL. The cultures were moved to an aerobic incubator for 6 h prior to harvesting to mimic conditions of the transwell experiments. Cultures were clarified by centrifugation at 7,000 × *g* for 30 min. The supernatant was filter sterilized (0.2-μm pore size polyethersulfone [PES] membrane) and then concentrated by ultrafiltration at 4°C using a Millipore stirred ultrafiltration apparatus (Millipore-Sigma, Burlington, MA). Concentrated supernatants were then ultracentrifuged at 100,000 × *g* for 2 h at 4°C to pellet crude OMVs, and the pellets were resuspended in 45% OptiPrep (Sigma-Aldrich) density gradient medium in HEPES buffer, overlaid with a continuous OptiPrep density gradient (45% to 15%), and centrifuged at 100,000 × *g* for 16 h. The fraction(s) containing the OMVs was suspended in HEPES buffer and then centrifuged at 100,000 × *g* for 2 h. The resultant purified OMV pellet was resuspended in a minimal volume of HEPES. Particle enumeration and size distribution were determined by NanoSight NS300 (Malvern Panalytical, Malvern, UK). The concentration of OMVs was normalized, and the OMVs were added directly to THP-1 cells at a ratio of 1,000 particles per host cell, as previously described (64).

### Statistical analysis.

Immunoassay data were collected, and both descriptive and comparative statistical analyses were performed with GraphPad 8.0 (GraphPad, San Diego, CA), using the nonpaired *t* test with Welch’s correction for unequal variances; the significance level was set at 95% (*P* < 0.05) for all analyses. Data were obtained from at least three independent experiments.

### Data availability.

Raw sequencing data are available in the NCBI Sequence Read Archive (SRA) under accession number PRJNA781251.
