# Supplementary material for: Characterization of a Bacterial Kinase That Phosphorylates Dihydrosphingosine to Form dhS1P
Source: Microbiol Spectr. 2022 Mar 14;10(2):e00002-22. doi: 10.1128/spectrum.00002-22 (PMC9045371; doi:10.1128/spectrum.00002-22)
Supplement: SUPPLEMENTAL FILE 1 — Supplemental material. Download SPECTRUM00002-22_Supp_1_seq13.pdf, PDF file, 2 MB [file spectrum00002-22_supp_1_seq13.pdf]

Table S1. Primers used in this study

Primers Name	Sequence	To construct
**PG1348_upF**	AAGCTTATTTCGATCGGTTATATC	ΔPG1348::ermR
**PG1348_upR**	TCTTTTTTGTCATAGCTATGCAAGAGTTAATCTTG	ΔPG1348::ermR
**PG1348_ermF**	TTGCATAGCTATGACAAAAAAGAAATTGCC	ΔPG1348::ermR
**PG1348_ermR**	GGTGGGACTTCTACGAAGGATGAAATTTTTC	ΔPG1348::ermR
**PG1348_dnF**	TCCTTCGTAGAAGTCCCACCTCTTTCTAAAAAC	ΔPG1348::ermR
**PG1348_dnR**	TCAGTCGATTTCGTTCGG	ΔPG1348::ermR
**PG1348_upcheck**	AAGCAAACGAATCGCTCTTGT	Sequencing
**PG1348_dncheck**	CGCATCAATGATTCAATCACC	Sequencing
**groESpro-BamHI-F**	cgcGGATCCATAGATGCCCTGCTCCCT	pTCOW-PG1348
**groES-PG1348-F**	TAACAATAACAAACCAAGCAACAATGAAGATTCTAGCCATTATCAAT	pTCOW-PG1348
**groES-PG1348-R**	ATTGATAATGGCTAGAATCTTCATTGTTGCTTGGTTTGTTATTGTTA	pTCOW-PG1348
**PG1348dn-SalI-R**	CgcGTCGACTCAAGTGTAGGTTTCAGTAGCA	pTCOW-PG1348

Supplemental Table 2. Differential gene expression in the W83 ΔPG1348 mutant compared to the parent strain.

	Old Name	Annotation	logFC	FDR
PG_RS05930	PG1348	diacylglycerol kinase family lipid kinase	-4.0376764	8.4E-136
PG_RS04360	PG0987	DUF4252 domain-containing protein	-2.0989729	6.634E-69
PG_RS04350	PG0985	sigma-70 family RNA polymerase sigma factor	-1.6357892	4.604E-57
PG_RS04355	PG0986	hypothetical protein	-1.7017489	8.247E-38
PG_RS02460	PG0555	Histone-like bacterial DNA-binding protein	1.204769	1.661E-14
hcp	PG0893	hydroxylamine reductase	1.072118	2.447E-12
htpG	PG0045	molecular chaperone HtpG	-1.1059228	6.637E-12
PG_RS02420	PG0541	hypothetical protein	1.1038002	1.287E-05
PG_RS10105	PG0524	DUF1661 domain-containing protein	1.1185827	3.615E-05
PG_RS00230	PG0050	IS4 family transposase	-1.4996494	0.0005363

Supplemental Table 3. Differential gene expression in W83Δ1348 compared to W83Δ1780 mutant. Specifically, genes not found to be differentially expressed when compared to the parent strain W83. PG1348 and PG1780 are provided for reference.

Name	Old Name	Annotation	Δ1348-Δ1780	
			logFC	FDR
PG_RS00055	PG0010	ATP-dependent Clp protease ATP-binding subunit	-1.125390081	1.77437E-06
trxA	PG0034	thioredoxin	1.264215462	1.72574E-05
PG_RS00495	PG0106	glycosyltransferase	1.027272917	1.09461E-05
PG_RS00510	PG0110	glycosyltransferase	1.157104441	1.69278E-07
PG_RS00515	PG0111	capsular polysaccharide biosynthesis protein	1.102054613	1.0896E-06
PG_RS00525	PG0113	AAC(3) family N-acetyltransferase	1.015309879	1.19347E-08
PG_RS00535	PG0115	serine acetyltransferase	1.111557463	2.22125E-11
PG_RS00545	PG0117	hypothetical protein	1.007823734	7.95548E-08
PG_RS00550	PG0118	glycosyltransferase family 2 protein	1.392893842	5.56634E-12
PG_RS00555	PG0119	WecB/TagA/CpsF family glycosyltransferase	1.059378553	1.96159E-10
secG		preprotein translocase subunit SecG	1.077228615	6.26114E-05
PG_RS01100	PG0241	hypothetical protein	-1.245074795	4.91833E-06
PG_RS01120	PG0246	hypothetical protein	1.055768651	4.42984E-05
PG_RS01255	PG0280	ABC transporter permease	-1.111658966	1.0395E-11
PG_RS01260	PG0281	FtsX-like permease family protein	-1.394558096	1.50963E-16
PG_RS01270	PG0283	HlyD family efflux transporter periplasmic adaptor	-1.134610787	1.88643E-08
PG_RS01520	PG0339	hypothetical protein	1.560092912	1.21809E-10
PG_RS01810		hypothetical protein	1.276766997	2.24367E-18
PG_RS01880	PG0423	hypothetical protein	1.046998269	3.28194E-06
PG_RS01995	PG0448	hypothetical protein	-1.070131454	4.91634E-06
lptC	PG0450	LPS export ABC transporter periplasmic protein LptC	-1.007210248	1.41156E-08
PG_RS02025	PG0456	phosphotransferase	1.042598017	2.18085E-05
PG_RS02100		DUF4248 domain-containing protein	1.429671788	3.03318E-07
PG_RS02140	PG0482	DUF5606 domain-containg protein	-1.154293512	1.61298E-07
PG_RS02300	PGt09		1.224403506	8.84197E-10
groL	PG0520	chaperonin GroEL	-1.501916588	1.8834E-08
PG_RS02310	PG0521	co-chaperone GroES	-1.301358129	7.96624E-11
PG_RS02415	PG0540	efflux RND transporter permease subunit	1.018335141	9.98873E-09
PG_RS02465		hypothetical protein	1.089012515	6.03711E-07
PG_RS02470	PG0556	hypothetical protein	1.142170561	1.43428E-09
PG_RS02475	PG0557	hypothetical protein	1.060738436	1.03403E-09
PG_RS02500	PG0563	hypothetical protein	1.048188366	3.1345E-15
PG_RS02505	PG0564	hypothetical protein	1.251494651	4.5708E-13

PG_RS02510	PG0565	hypothetical protein	1.201140176	6.68477E-09
PG_RS02985	PG0679	TolC family protein	-1.084802467	2.00061E-05
PG_RS03500	PG0798	IS982-like element IS195 family transposase	1.138343453	3.95117E-08
PG_RS03650	PG0832	nucleoid-associated protein	1.582275271	3.39874E-10
PG_RS03655	PG0833	DUF3732 domain-containing protein	1.374951318	3.44895E-07
PG_RS03660		hypothetical protein	1.187149424	3.16166E-09
PG_RS03665	PG0834	DUF4297 domain-containing protein	1.166827189	1.42876E-05
PG_RS03670		helix-turn-helix transcriptional regulator	1.159211786	2.90347E-09
PG_RS03705	PG0843	DUF3853 family protein	1.330270902	1.41426E-12
PG_RS03710	PGt19		1.485102604	6.17447E-13
PG_RS03765	PG0856	hypothetical protein	1.356842235	1.11873E-12
PG_RS03770	PG0857	helix-turn-helix transcriptional regulator	1.242935668	4.07325E-17
PG_RS03775	PG0858	conserved hypothetical protein	1.269829754	2.42447E-18
PG_RS03800		class I SAM-dependent DNA methyltransferase	1.113925544	4.17585E-16
PG_RS03845	PG0872	helix-turn-helix domain-containing protein	1.005790355	1.29833E-06
PG_RS03850		hypothetical protein	1.26524212	9.38949E-11
PG_RS03855	PG0873	hypothetical protein	1.373909326	4.54387E-13
PG_RS03860	PG0874	site-specific integrase	1.111171253	4.78103E-14
PG_RS03990	PG0906	hypothetical protein	-1.409613337	1.05322E-07
PG_RS04280	PGt22		1.033458432	6.57903E-10
PG_RS04845	PGt25		1.205833869	6.09897E-06
PG_RS04850	PGt26		1.681249494	2.98778E-15
PG_RS04895	PG1107	hypothetical protein	1.023328373	1.13842E-05
PG_RS05300	PG1202	hypothetical protein	1.128385157	1.522E-05
dnaK	PG1208	molecular chaperone DnaK	-1.682851584	4.51499E-11
PG_RS05630	PG1277	nucleotide sugar dehydrogenase	1.038094668	1.81755E-06
queF	PG1347	NADPH-dependent 7-cyano-7-deazaguanine reductase	1.037499078	7.7522E-16
PG_RS05930	PG1348	diacylglycerol kinase family lipid kinase	-4.417739812	1.39E-158
PG_RS06295	PGt33		1.04787658	6.56613E-10
PG_RS06305	PG1436	ATP-binding protein	1.043584033	2.68464E-07
PG_RS06400	PG1458	DUF1896 domain-containing protein	1.177311573	1.7474E-11
PG_RS06450		hypothetical protein	1.065241435	1.59393E-05
PG_RS06565	PG1491	hypothetical protein	1.172140965	3.99443E-05
PG_RS06570	PG1492	GLPGLI family protein	1.161337473	1.32245E-06
PG_RS06575	PG1493	hypothetical protein	1.130940726	4.70571E-06
PG_RS06615	PG1501	TetR/AcrR family transcriptional regulator	1.177009421	8.63119E-07
PG_RS06635	PG1504	NAD(P)-dependent oxidoreductase	1.722336398	0.091386618
PG_RS06655	PG1508	hypothetical protein	1.614791612	0.091145325

PG_RS06670	PG1513	phosphoribosyltransferase	1.455514095	1.99147E-14
PG_RS06740	PG1529	hypothetical protein	1.142707687	2.98028E-06
PG_RS06745	PG1530	GTP pyrophosphokinase	1.135269787	3.07977E-07
PG_RS06755	PG1532	helix-turn-helix domain-containing protein	1.01663753	1.12142E-08
PG_RS06855	PG1554	hypothetical protein	-1.677492765	3.79705E-06
PG_RS06980	PG1586	tetratricopeptide repeat protein	-1.001183623	1.21859E-07
PG_RS07000	PG1591	type I restriction-modification system subunit R	1.349586194	1.69752E-06
PG_RS07005	PGt35		1.226440569	5.03425E-13
PG_RS07420	PG1683	alpha-amylase	-1.206616797	5.5814E-13
PG_RS07810	PG1775	nucleotide exchange factor GrpE	-1.060290389	2.1199E-14
PG_RS07830	PG1779	O-acetyl-ADP-ribose deacetylase	-1.104426644	2.16581E-15
PG_RS07835	PG1780	serine palmitoyl transferase	2.678402624	2.63E-61
PG_RS08035	PGt42		1.140514147	2.80322E-09
PG_RS08040	PG1823	PorT family protein	-1.19122458	4.54077E-05
PG_RS08060	PG1828	hypothetical protein	1.369248822	1.82503E-05
PG_RS08285	PG1890	hypothetical protein	1.334976938	1.36591E-11
PG_RS08290	PG1891	hypothetical protein	1.024373659	9.35283E-10
PG_RS08300	PG1892	hypothetical protein	1.453040124	3.44948E-12
PG_RS08720	PG1975	hypothetical protein	1.307445879	1.45647E-08
cmr6	PG1983	type III-B CRISPR module RAMP protein Cmr6	1.183887669	7.7522E-16
cas2	PG2013	CRISPR-associated endonuclease Cas2	1.039235627	2.00137E-11
PG_RS09055	PG2050	hypothetical protein	1.031458221	0.000289428
PG_RS09085	PGt44		1.175016865	1.15646E-09
PG_RS09150	PG2069	SDR family oxidoreductase	1.008281898	6.75709E-05
PG_RS09545	PGt48		1.204535741	3.193E-16
PG_RS09665	PGt49		1.172755446	3.97041E-08
PG_RS09830	PG2212	DUF1661 domain-containing protein	1.105099475	1.24673E-10
PG_RS09870	PGt53		1.258174837	1.24673E-10
PG_RS09895	PG2224	hypothetical protein	1.118742548	2.63872E-16
PG_RS10040		DUF1661 domain-containing protein	1.115776117	0.000152037
PG_RS10175		hypothetical protein	1.700142436	1.70495E-05
PG_RS10215	PG0799	hypothetical protein	1.090692927	5.29184E-07
PG_RS10250		glycine cleavage system H protein	1.639898477	1.33992E-16
PG_RS10275		hypothetical protein	1.13265497	5.89645E-13
PG_RS10550		DUF1661 domain-containing protein	1.417886303	3.18268E-11
PG_RS10615	PG1722	DUF1661 domain-containing protein	1.033469433	2.51521E-10
PG_RS10890		DUF1661 domain-containing protein	1.102209679	7.36056E-05
PG_RS10910		DUF1661 domain-containing protein	1.282819188	3.5514E-09
PG_RS10920		DUF1661 domain-containing protein	1.228074092	0.034667706

PG_RS11000	DUF1661 domain-containing protein	1.118746822	0.000102253
PG_RS11005	DUF1661 domain-containing protein	1.094204362	2.15743E-05
PG_RS11010	hypothetical protein	1.486517573	7.009E-05
PG_RS11175	hypothetical protein	1.297006932	6.24183E-06
PG_RS11265	hypothetical protein	-1.023422737	0.000527793
PG_RS11315	hypothetical protein	1.065218064	7.71636E-10
PG_RS11335	hypothetical protein	1.047494077	1.24706E-07
PG_RS11370	hypothetical protein	1.14997216	4.38426E-08

Supplemental Table 4. Differential gene expression in the sphingolipid null W83 Δ1780 mutant compared to the parent strain.

Name	Old Name	Annotation	logFC	FDR
PG_RS08305		hypothetical protein	-1.02898	4.1107E-05
PG_RS08310		hypothetical protein	-1.17152	7.0318E-06
PG_RS08780		hypothetical protein	-1.52949	1.3894E-14
PG_RS10060		DUF1661 domain-containing protein	-1.05418	0.00032472
PG_RS10395		hypothetical protein	-1.16903	0.05052901
PG_RS10460		DUF1661 domain-containing protein	-1.40184	6.0743E-08
PG_RS10520		hypothetical protein	-1.09239	7.1872E-06
PG_RS10855		DUF1661 domain-containing protein	-1.31059	6.2058E-05
PG_RS11190		DUF1661 domain-containing protein	-1.74319	9.5383E-11
PG_RS11325		hypothetical protein	-1.74218	0.10221991
porV	PG0027	type IX secretion system outer membrane channel PorV	1.443115	1.3186E-07
PG_RS00130	PG0028	2-C-methyl-D-erythritol 2 2C4-cyclodiphosphate synthase	1.068376	4.7649E-08
PG_RS00230	PG0050	IS4 family transposase	-1.47995	0.00027484
PG_RS00295	PG0064	CusA/CzcA family heavy metal efflux RND transporter	1.019635	2.8233E-10
PG_RS11255	PG0161	hypothetical protein	1.637266	1.6136E-16
PG_RS00975	PG0214	sigma-70 family RNA polymerase sigma factor	2.437099	2.9549E-21
PG_RS00980	PG0215	hypothetical protein	2.094592	1.4952E-17
PG_RS00985	PG0216	DUF4252 domain-containing protein	2.07468	1.3635E-20
PG_RS00990	PG0217	hypothetical protein	2.17405	4.807E-18
PG_RS00995	PG0218	hypothetical protein	2.102884	1.3812E-15
PG_RS01285	PG0287	type IX secretion system membrane protein PorP/SprF	1.32616	5.7059E-12
PG_RS01290	PG0288	SUMF1/EgtB/PvdO family nonheme iron enzyme	1.331999	2.287E-09
gldL	PG0289	gliding motility protein GldL	1.258083	1.8151E-10
gldM	PG0290	gliding motility protein GldM	1.082608	4.5343E-07
gldN	PG0291	gliding motility protein GldN	1.281177	2.0677E-07
PG_RS01450	PG0323	cupin domain-containing protein	1.079705	4.1195E-06
PG_RS01865	PG0419	DUF2807 domain-containing protein	1.114121	3.3918E-05
PG_RS02400	PG0537	aminoacyl-histidine dipeptidase	-2.5364	2.9527E-27
PG_RS02450	PG0549	IS5-like element ISPg8 family transposase	-1.95271	3.7578E-30
PG_RS02460	PG0555	Histone-like bacterial DNA-binding protein	-1.01596	0.00031051
PG_RS02690	PG0607	hypothetical protein	1.192587	0.00110638
PG_RS02720	PG0616	hypothetical protein	-1.43934	0.00014223
PG_RS10165	PG0617	hypothetical protein	-1.21753	9.5383E-11
PG_RS03180	PG0726	PEGA domain-containing protein	-2.03792	1.0026E-10
PG_RS04015	PG0914	hypothetical protein	-1.33343	3.7529E-09
PG_RS04350	PG0985	sigma-70 family RNA polymerase sigma factor	-1.8352	4.2739E-34
PG_RS04355	PG0986	hypothetical protein	-1.59554	3.8234E-23
PG_RS04360	PG0987	DUF4252 domain-containing protein	-2.33274	1.2397E-36
PG_RS04645	PG1055	thiol protease	2.846891	0.00026098

clpB	PG1118	ATP-dependent chaperone ClpB	1.070476	8.7005E-06
PG_RS05215	PG1177	IS5 family transposase	-1.06253	0.05394282
PG_RS05265	PG1190	D-2-hydroxyacid dehydrogenase	-1.07276	3.0325E-05
pruA	PG1269	L-glutamate gamma-semialdehyde dehydrogenase	-1.49007	0.00043395
PG_RS05610	PG1270	amidinotransferase	-1.51508	0.00017408
rocD	PG1271	ornithine--oxo-acid transaminase	-1.46175	0.00073733
PG_RS05835	PG1326	hemagglutinin	-1.00445	7.2458E-09
PG_RS06055	PG1374	T9SS type A sorting domain-containing protein	1.25144	4.3252E-06
PG_RS06520	PG1482	DUF4133 domain-containing protein	-1.23541	0.00705727
PG_RS06580	PG1494	DUF3945 domain-containing protein	-1.06268	1.1487E-10
PG_RS06640	PG1505	radical SAM protein	-1.70276	0.1368797
PG_RS06660	PG1509	HAD family hydrolase	-1.57869	0.09875182
PG_RS06665	PG1512	DNA helicase	-1.50986	0.00066921
PG_RS06675	PG1514	iron-containing alcohol dehydrogenase	-1.19213	3.0993E-06
PG_RS06680	PG1515	ribulose-bisphosphate carboxylase	-1.23209	9.6671E-07
PG_RS06685	PG1516	hypothetical protein	-1.13609	3.3697E-05
PG_RS10525	PG1527	helix-turn-helix domain-containing protein	1.157073	0.00187708
PG_RS07170	PG1625	hypothetical protein	1.059251	0.00015759
PG_RS07175	PG1626	transporter	1.14053	1.2075E-05
PG_RS07195	PG1634	hypothetical protein	1.048181	8.5929E-07
PG_RS07415	PG1682	glycosyltransferase	1.163496	1.0722E-14
PG_RS07425	PG1684	hypothetical protein	1.57735	1.6195E-08
PG_RS07835	PG1780	pyridoxal phosphate-dependent aminotransferase	-2.8119	3.0166E-52
udk	PG1781	uridine kinase	1.305555	6.7388E-09
PG_RS07920	PG1795	hypothetical protein	-1.4174	3.2307E-06
PG_RS07930	PG1798	T9SS type A sorting domain-containing protein	-1.25218	7.9904E-05
PG_RS08080	PG1835	DUF4270 family protein	1.002664	9.7455E-05
PG_RS08315	PG1893	hypothetical protein	-1.00436	0.00031319
PG_RS08400	PG1908	GLPGLI family protein	-1.23376	0.00014194
PG_RS08580	PG1945	hypothetical protein	1.128931	2.0332E-08
PG_RS08585	PG1946	metal ABC transporter permease	1.049348	2.5606E-08
PG_RS08690	PG1967	tetratricopeptide repeat protein	1.073187	1.3911E-08
cas2	PG1981	CRISPR-associated endonuclease Cas2	-1.36552	6.101E-10
cas1	PG1982	CRISPR-associated endonuclease Cas1 type III-B	-1.07952	2.7714E-10
PG_RS08750	PG1984	hypothetical protein	-1.45284	3.3506E-16
cmr4	PG1985	type III-B CRISPR module RAMP protein Cmr4	-1.23419	1.2119E-13
cmr3	PG1986	type III-B CRISPR module-associated protein Cmr3	-1.49734	1.8935E-14
cas10	PG1987	type III-B CRISPR-associated protein Cas10/Cmr2	-1.81276	1.6966E-17
PG_RS08770	PG1988	hypothetical protein	-2.06658	8.5539E-22
PG_RS08775	PG1989	hypothetical protein	-1.90426	2.0387E-26
cas1b	PG2014	type I-B CRISPR-associated endonuclease Cas1	-1.39433	6.7084E-18
cas4	PG2015	CRISPR-associated protein Cas4	-1.39863	1.2945E-16
PG_RS08890	PG2016	CRISPR-associated helicase/endonuclease Cas3	-1.5948	1.8723E-09

cas7p	PG2017	type I-PGING CRISPR-associated protein Cas7/Csp1	-1.645	2.7982E-08
cas8c	PG2018	type I-PGING CRISPR-associated protein Cas8c/Csp2	-1.64102	1.562E-09
cas5p	PG2019	type I-PGING CRISPR-associated protein Cas5p	-1.76591	3.4592E-19
cas6	PG2020	CRISPR-associated endoribonuclease Cas6	-1.46716	1.2119E-13
PG_RS09310	PG2100	T9SS type A sorting domain-containing protein	3.253986	4.4793E-05
PG_RS09315	PG2101	hypothetical protein	3.958014	9.7471E-06
PG_RS09320	PG2102	T9SS type A sorting domain-containing protein	4.228356	7.6167E-06
PG_RS09335	PG2106	PorT family protein	1.08783	0.00081061
PG_RS09760	PG2199	ABC-F family ATP-binding cassette domain-containing protein	1.028491	6.4623E-07
rrf	PG5SB		-3.73522	0.00424729
PG_RS00035	PGt01		-1.07461	0.00032513
PG_RS03385	PGt16		-1.05741	0.0002116
PG_RS04335	PGt23		-1.43655	4.752E-07
PG_RS05460	PGt28		-1.07257	2.4794E-05
PG_RS07865	PGt40		-1.02549	1.4234E-06
PG_RS07870	PGt41		-1.14908	1.9401E-05
PG_RS09325	PGt47		-1.11386	4.1195E-06

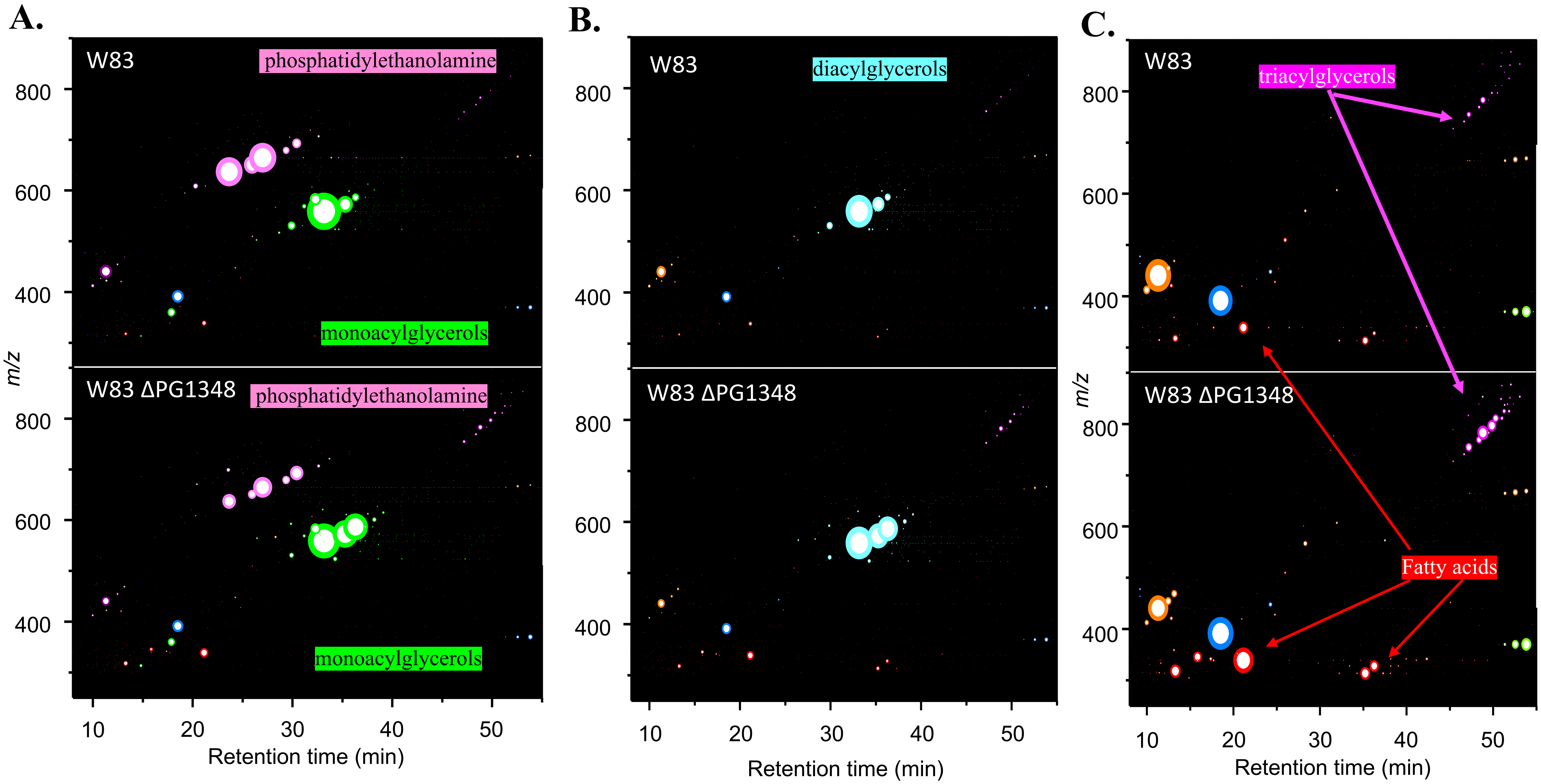

Fig. S1. Bubble plots of lipids (non-sphingolipids) and fatty acids in *P. gingivalis* cells. Size of bubble and retention times show the amount and different lipid subtypes, respectively. **A**. The parent strain W83 contains higher levels of phosphatidylethanolamine and less monacylglycerols than the ΔPG1348 mutant. **B**. The ΔPG1348 mutant cells contain more diacylglycerols, and (**C**) more triacyglycerols, as well as free fatty acids.

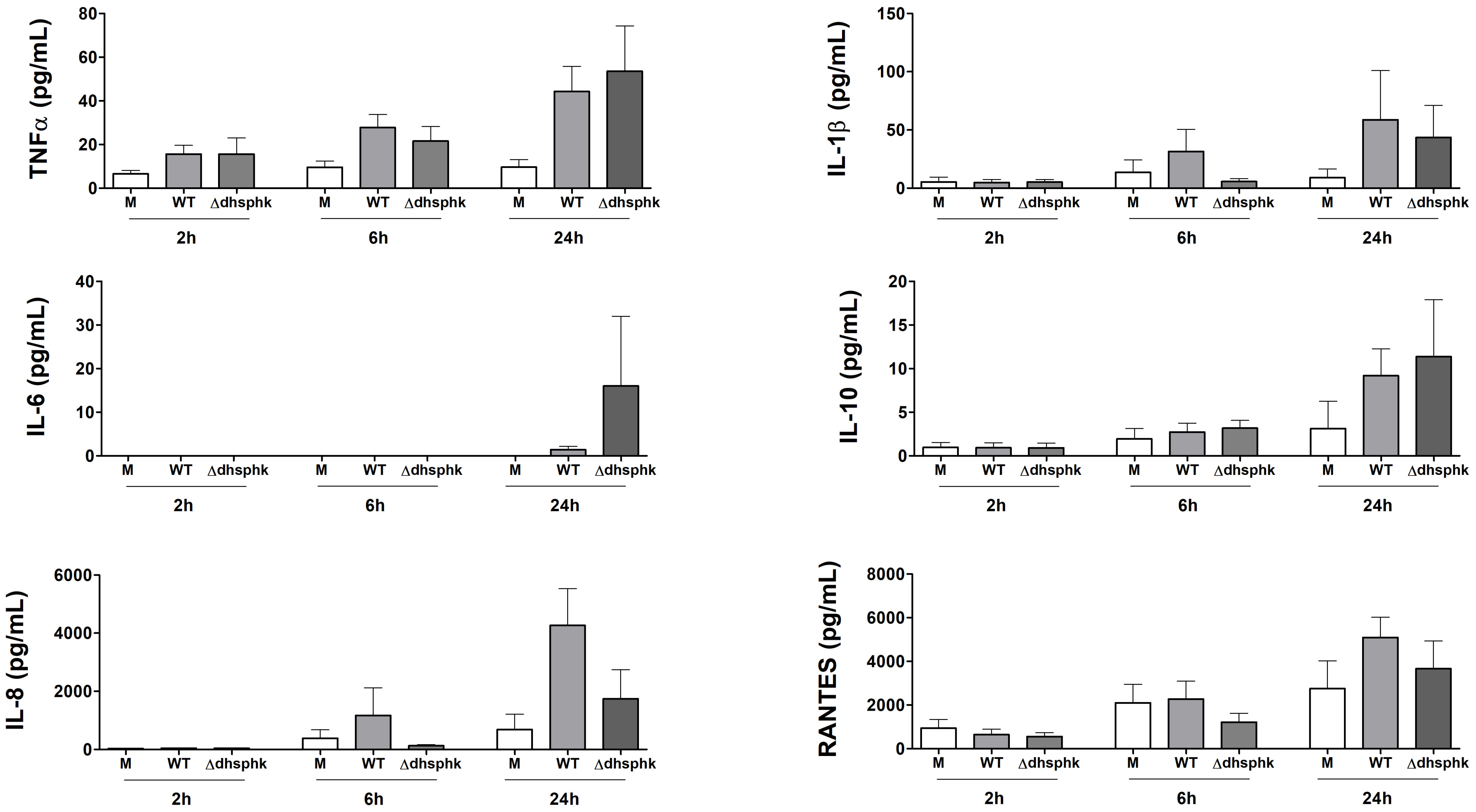

Figure S2. Impact of *dhSphK1* on cytokines and chemokines response. Human macrophage-like THP-1 cells were directly cultured with **purified OMVs** (1,000 particles/cell) from *P. gingivalis* W83 (WT) and the Δ*dhsphK1* mutant. Supernatant fluids were collected at 2, 6, and 24 h, and the levels of TNF-α, IL-1b, IL-6, IL-10, RANTES, and IL-8 were measured by multiplex immunoassay. Medium alone (M) served as unchallenged (negative) controls.

A.

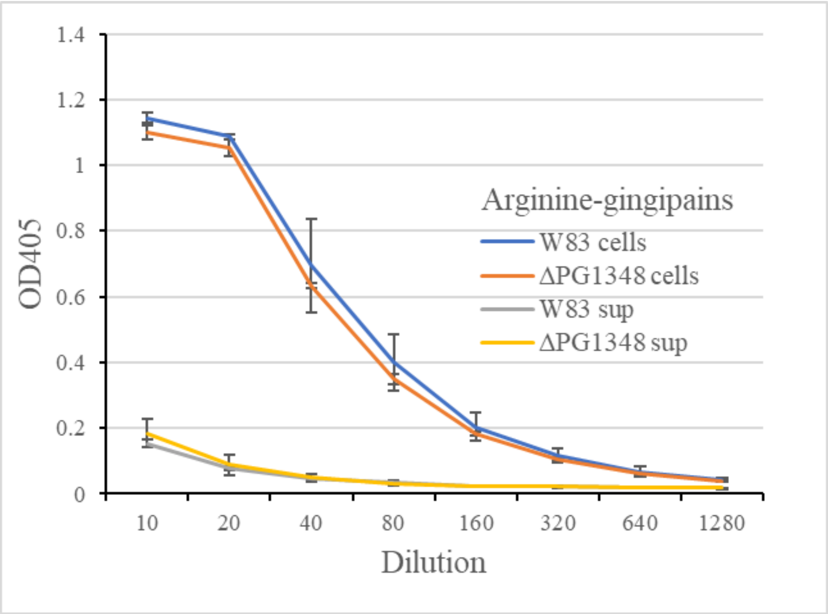

B.

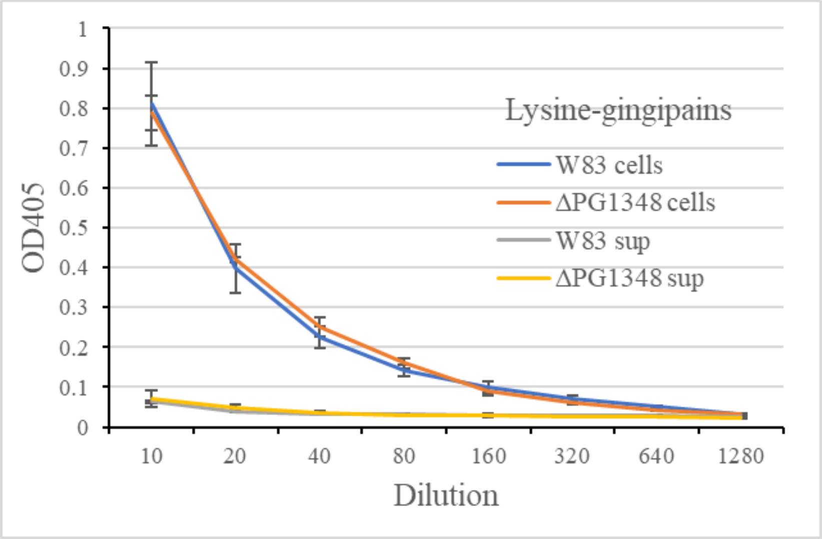

Figure S3. Gingipain assays did not detect any difference in cell associated or secreted (sup) gingipain activity in the ΔPG1348 mutant when compared to the parent strain W83. This aligns with no defect in the levels of A-LPS, which is known to anchor the gingipains to the cell surface.

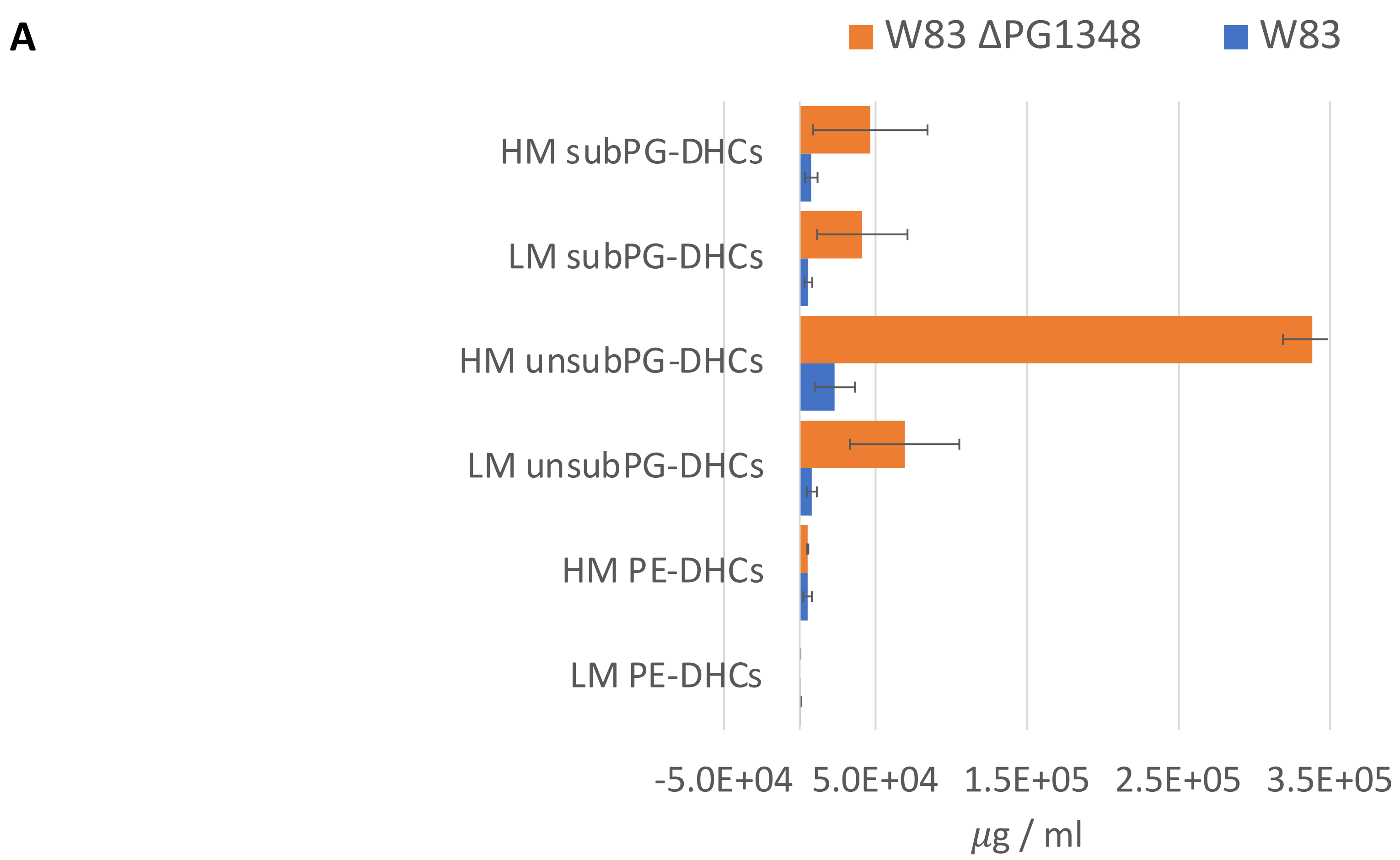

Fig. S4 (**A**.) LCMSMS lipid analysis showed higher relative levels of PG-DHCs in the ΔPG1348 mutant, similar to QTQ analysis shown in Fig 4.

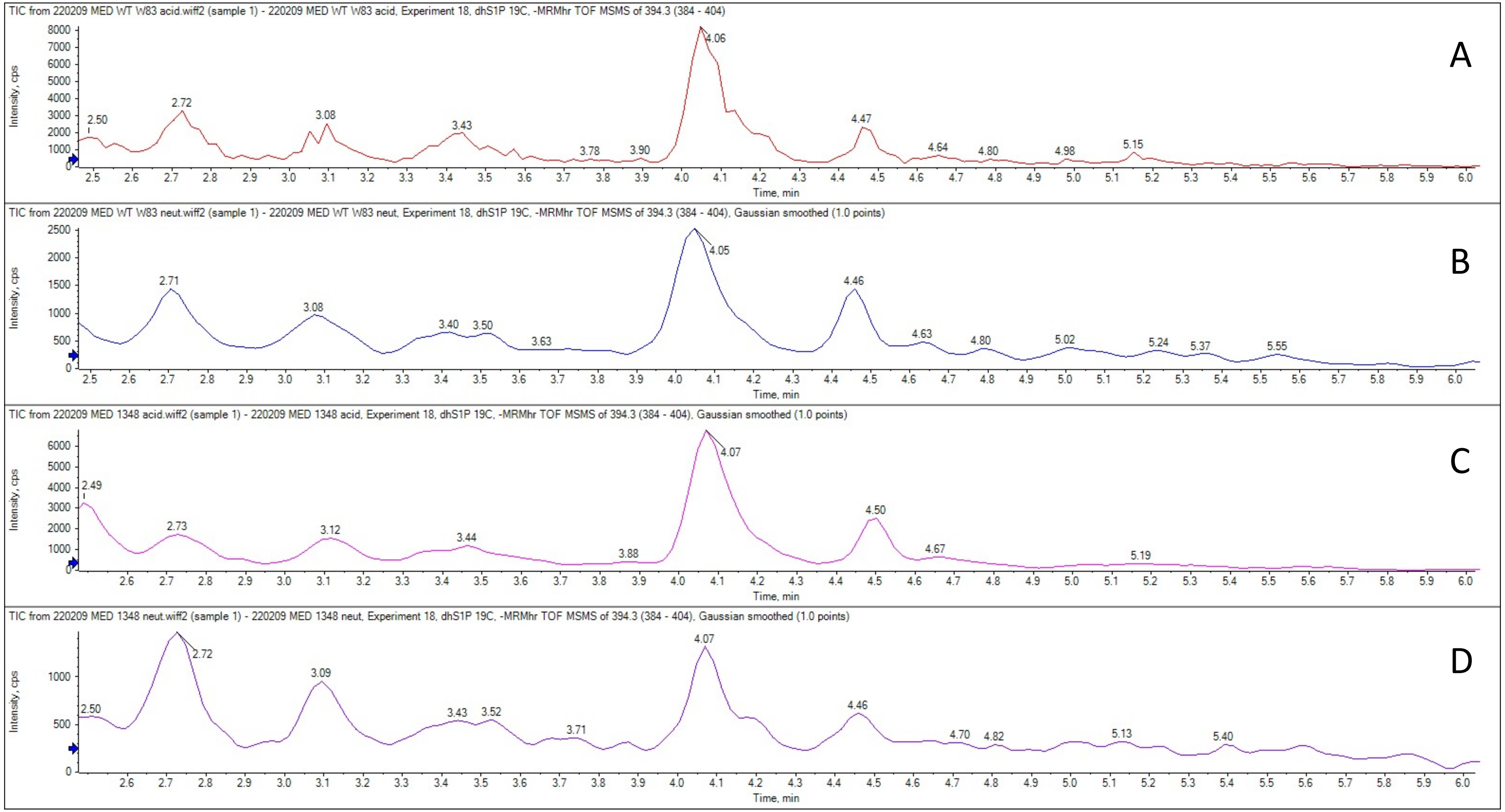

Figure S5. Detection of C19 dhS1P by LC-MS/MS. Samples were processed as described in the Materials and Methods. The acidic and neutral extracts of WT W83 and the 1348 mutant were evaluated for the molecular negative ion (m/z 394.3) consistent with the exact mass of $C_{19:0}$ dhS1P (394.2722). The acidic (A) and the neutral (B) extracts of WT W83 showed molecular ion scans consistent with the presence of $C_{19:0}$ dhS1P. The acidic (C) and neutral (D) extracts of the PG1348 mutant also demonstrated molecular ions consistent with the presence of $C_{19:0}$ dhS1P (Time 4.0). The same method was used to evaluate a synthetic standard of $C_{18:0}$ dhS1P and revealed a molecular ion mass of *m/z* 380.2848 (exact mass 380.2566, data not shown).
